# Metabolomics and metabolic pathway networks from human colorectal cancers, adjacent mucosa, and stool

**DOI:** 10.1186/s40170-016-0151-y

**Published:** 2016-06-06

**Authors:** Dustin G. Brown, Sangeeta Rao, Tiffany L. Weir, Joanne O’Malia, Marlon Bazan, Regina J. Brown, Elizabeth P. Ryan

**Affiliations:** Department of Environmental and Radiological Health Sciences, Colorado State University, 200 West Lake Street, 1680 Campus Delivery, Fort Collins, CO 80523 USA; Department of Clinical Sciences, Colorado State University, Fort Collins, CO 80523 USA; Department of Food Science and Human Nutrition, Colorado State University, Fort Collins, CO 80523 USA; University of Colorado Health-North, Fort Collins, CO 80522 USA; Division of Medical Oncology, University of Colorado School of Medicine, Aurora, CO 80045 USA

**Keywords:** Metabolomics, Colorectal, Cancer, Stool, Metabolites, Tumor, Colon mucosa, Metabolic pathways

## Abstract

**Background:**

Colorectal cancers (CRC) are associated with perturbations in cellular amino acids, nucleotides, pentose-phosphate pathway carbohydrates, and glycolytic, gluconeogenic, and tricarboxylic acid intermediates. A non-targeted global metabolome approach was utilized for exploring human CRC, adjacent mucosa, and stool. In this pilot study, we identified metabolite profile differences between CRC and adjacent mucosa from patients undergoing colonic resection. Metabolic pathway analyses further revealed relationships between complex networks of metabolites.

**Methods:**

Seventeen CRC patients participated in this pilot study and provided CRC, adjacent mucosa ~10 cm proximal to the tumor, and stool. Metabolomes were analyzed by gas chromatography-mass spectrometry (GC/MS) and ultra-performance liquid chromatography-mass spectrometry (UPLC-MS/MS). All of the library standard identifications were confirmed and further analyzed via MetaboLync^TM^ for metabolic network interactions.

**Results:**

There were a total of 728 distinct metabolites identified from colonic tissue and stool matrices. Nineteen metabolites significantly distinguished CRC from adjacent mucosa in our patient-matched cohort. Glucose-6-phosphate and fructose-6-phosphate demonstrated 0.64-fold and 0.75-fold lower expression in CRC compared to mucosa, respectively, whereas isobar: betaine aldehyde, N-methyldiethanolamine, and adenylosuccinate had 2.68-fold and 1.88-fold higher relative abundance in CRC. Eleven of the 19 metabolites had not previously been reported for CRC relevance. Metabolic pathway analysis revealed significant perturbations of short-chain fatty acid metabolism, fructose, mannose, and galactose metabolism, and glycolytic, gluconeogenic, and pyruvate metabolism. In comparison to the 500 stool metabolites identified from human CRC patients, only 215 of those stool metabolites were also detected in tissue. This CRC and stool metabolome investigation identified novel metabolites that may serve as key small molecules in CRC pathogenesis, confirmed the results from previously reported CRC metabolome studies, and showed networks for metabolic pathway aberrations. In addition, we found differences between the CRC and stool metabolomes.

**Conclusions:**

Stool metabolite profiles were limited for direct associations with CRC and adjacent mucosa, yet metabolic pathways were conserved across both matrices. Larger patient-matched CRC, adjacent non-cancerous colonic mucosa, and stool cohort studies for metabolite profiling are needed to validate these small molecule differences and metabolic pathway aberrations for clinical application to CRC control, treatment, and prevention.

**Electronic supplementary material:**

The online version of this article (doi:10.1186/s40170-016-0151-y) contains supplementary material, which is available to authorized users.

## Background

Metabolomics has helped cancer research to elucidate specific biomarkers of disease that facilitate personalized therapies and may improve clinical outcomes non-invasively following primary or secondary diagnoses [1]. Colorectal cancer (CRC) is the third leading cause of cancer-related death in the USA [2] and while there are many screening techniques (i.e., colonoscopy, double contrast barium enema, immunochemical-based fecal occult blood test, and serum CEA test [3–5]), high throughput and sensitive molecular tools are needed to support identification of novel biomarkers for metabolic pathway aberrations. Metabolomics is an emerging analytical tool that can be described as the systematic study of the entire profile of small molecules in a clinical sample that are detected using mass spectrometry. Metabolites from a multitude of matrices, such as serum, urine, stool, and tissue, represent the downstream functional products of gene expression and protein synthesis and include environmental exposures and microbial metabolism, all of which influence CRC processes [6]. There remains a gap in knowledge for the spectrum of metabolic pathway alterations that co-exist in the CRC tumor microenvironment which merits a non-targeted, global metabolomics approach [7].

Chan et al. reported 31 metabolites in 2009 that were differentially expressed in paired CRC and adjacent mucosal samples, with glucose levels that were ~67 % higher in the mucosa [8]. In 2014, Qiu et al. identified 15 significantly altered metabolites in matched surgical specimens by comparing CRC and adjacent mucosa from 3 different hospitals in China and 1 in the USA [9]. These metabolite signatures were then used to predict the rate of recurrence and survival in patients after treatment [9]. Most CRCs originate from polyps that exhibit metabolic alterations due to distinct mutations that allow them to replicate uncontrollably, evade host immunity, and become invasive (i.e., Warburg effect, *APC*/*KRAS*/*BRAF* gene mutations, MSI status) [10–12]. Metabolic fingerprints that could distinguish adjacent mucosa from CRC may reveal stages of pathogenesis, inform the frequency of follow-up screening, and response to preventive measures needed for improved prognosis [13, 14].

Paralleled investigations of CRC and adjacent mucosal tissues alongside patient-matched stools that reflect the tumor microenvironment are limited [3]. In the present pilot study, we determined metabolite profile differences between patient-matched CRC and adjacent mucosa and performed metabolic pathway analyses to examine correlations between metabolite expression. We hypothesized that identification of metabolite signatures of colonic tissue that are proximal to and within the CRC represent potential metabolic targets for secondary CRC control, prevention, and treatment. The overlapping metabolites from patient-matched stool and tissue metabolomes as aberrancies in metabolic pathway networks presented herein merit further interrogation for enhanced knowledge of CRC etiology and pathogenesis.

## Methods

### Ethics, consent, and permissions

Seventeen individuals with a scheduled colonic resection after a CRC diagnosis were recruited for this study. All patients provided informed, written consent and knew that data would be used for publication. The patient inclusion criteria for this study included not taking antibiotics prior to surgery (intravenous pre-operative antibiotics were admissible). Tissue and stool samples were collected from Poudre Valley Hospital (PVH; Fort Collins, CO, USA) and de-identified for personal information before processing at Colorado State University (protocol nos. 10-1006 and 10-1670H).

### Sample collection

Within 30 min of surgery, a 5-mm section of CRC (*n* = 16) and adjacent mucosal tissue samples that were ~10 cm proximal to the CRC (*n* = 17) were collected from resected colons in the PVH Pathology Lab. Human tissue samples were stored immediately at −80 °C following collection until processed for metabolomics. Stool samples were self-collected by patients just prior to surgery prep in pre-labeled, coded containers by the patients and stored at −80 °C immediately (*n* = 13). Collection was verified by the study technician who was present in the pathology lab and pre-op room for each resection. Patient-matched CRC, adjacent mucosa, and stool were collected for all individuals with the following exceptions: one patient was missing a CRC sample but had an adjacent mucosa and stool sample, and four patients provided mucosa and CRC but did not provide stool. All samples were shipped to Metabolon, Inc. (Durham, NC, USA) for metabolomics and multivariate statistical analyses. Table 1 shows human patient and tumor characteristics associated with tissue samples analyzed herein [15].

**Table 1:** Colorectal cancer patient and tumor characteristics

Age (years) [mean ± SD]	58.8 ± 13.8
Sex	
Males	13 (76 %)
Females	4 (24 %)
BMI (kg/m^2^) [mean ± SD]	29.3 ± 4.7
Tumor stage	
Tx/T0	1 (6 %)
Tis	1 (6 %)
T1	3 (18 %)
T2	3 (18 %)
T3	8 (46 %)
T4	1 (6 %)
Tumor location	
Cecum	1 (6 %)
Ascending	5 (31 %)
Descending	1 (6 %)
Sigmoid	6 (38 %)
Rectum	3 (19 %)
Tumor size (cm)	
0 < 2	6 (35 %)
2 < 4	5 (29 %)
4 < 6	3 (18 %)
6 < 8	3 (18 %)
Tumor grade	
Low	15 (94 %)
High	1 (6 %)

### Sample accessioning and preparation

Tissue and stool metabolite extractions for GC-MS and UPLC-MS/MS were completed by Metabolon Inc. Both platforms were chosen to provide broad, non-targeted detection of metabolites. Each sample was accessioned into the Metabolon Laboratory Information Management System (LIMS) and assigned a unique identifier associated with the original de-identified study code number. This identifier was used to track all sample handling, tasks, and results. All samples were maintained at −80 °C until analysis [16].

Samples were extracted using the automated MicroLab STAR® robotics system from Hamilton Company (Reno, NV, USA). A set of recovery standards, tridecanoic acid, chloro-phenylalanine, D6-cholesterol, and fluoro-phenylglycine was added prior to the first step in the extraction process for quality control purposes, with final extraction standard concentrations ranging from 2.5 to 25 μg/mL. Sample preparation was conducted using a methanol extraction to remove the protein fraction while allowing the maximum recovery of small molecules. Tissue samples were homogenized in water (5 μL/mg of sample) using a GenoGrinder 2000 bead grinder (Glen Mills, Clifton, NJ, USA), shaking for 5 min at 1000 strokes per minute at room temperature. Methanol extraction utilized five volumes of methanol (5:1 methanol/water) with vigorous shaking at room temperature for 2 min followed by centrifugation at 680 ×*g* for 3 min. The resulting methanol extract was divided into four fractions: one for analysis by UPLC-MS/MS with positive ion mode electrospray ionization and one for negative ion mode electrospray ionization, one for analysis by GC-MS, and one sample was reserved for backup. Samples were placed briefly on a TurboVap® from Zymark Corporation (Hopkinton, MA, USA) to remove the organic solvent. The samples were stored overnight under nitrogen before preparation for UPLC-MS/MS and each sample was dried under vacuum for a minimum of 18 h before derivatization for GC-MS analysis. Samples were analyzed in concert with several types of controls to allow instrument performance monitoring and aid chromatographic alignment.

### GC-MS analysis

Samples were derivatized under nitrogen using bistrimethyl-silyltrifluoroacetamide and separated on a 5 % diphenyl/95 % dimethyl polysiloxane-fused silica column (20 m × 0.18 mm ID; 0.18-μm film thickness) with helium as carrier gas and a temperature ramp from 60 to 340 °C in a 17.5-min period. Internal standards amylbenzene, 1-phenylhexane, 1-phenyloctane, 1-phenyldecane, 1-phenyldodecane, hexadecylbenzene, octadecylbenzene, tetradecylbenzene, and 2,6-di-tert-butyl-4-methylphenol were added to each sample (250 ng of each standard per sample). Samples were analyzed on a Thermo-Finnigan Trace DSQ fast-scanning single-quadrupole mass spectrometer using electro-impact ionization (EI) and operated at unit mass resolving power. The scan range was from 50–750 m/z.

### UPLC-MS/MS analysis

The UPLC-MS/MS portion of the platform was based on a Waters ACQUITY UPLC and a Thermo-Finnigan LTQ MS operated at nominal mass resolution, which consisted of an electrospray ionization (ESI) source and linear ion-trap (LIT) mass analyzer. The dried sample extract was reconstituted in acidic or basic UPLC-compatible solvents, each of which contained 11 to 13 injection standards at fixed concentrations [17]. One aliquot was analyzed using acidic positive ion-optimized conditions and the other using basic, negative ion-optimized conditions in two independent injections using separate dedicated columns (Waters UPLC BEH C18-2.1 × 100 mm, 1.7 μm). Extracts reconstituted in acidic conditions were gradient eluted using water and methanol containing 0.1 % formic acid, while the basic extracts, which also used water/methanol, contained 6.5 mM ammonium bicarbonate. The MS analysis alternated between MS and data-dependent MS/MS scans using dynamic exclusion and the scan range was from 80–1000 m/z.

### Data extraction and compound identification

Raw data was extracted and peak-identified as previously described [14]. Biochemical identifications were based on (1) retention index within a narrow RI window of the proposed identification, (2) accurate mass match to the library +/− 0.005 amu, and (3) the MS/MS forward and reverse scores between the experimental data and authentic standards. The MS/MS scores were based on a comparison of the ions present in the experimental spectrum to the ions present in the library spectrum. More than 3300 commercially available purified standard compounds have been acquired and registered into LIMS for distribution to both the UPLC-MS/MS and GC-MS platforms for determination of their analytical characteristics [16]. All detected metabolites were identified with level 1 or 2 (noted by an asterisk symbol) confidence of identification [18].

### Metabolomics statistical analysis

Metabolite profiles in CRC patients were quantified in terms of relative abundance and median scaled to 1. Following log transformation and imputation of missing values, if any, with the minimum observed value for each compound imputed, statistical analyses were performed to identify significant differences between experimental groups. Statistical analyses were performed in ArrayStudio (Omicsoft, Cary, NC, USA), R version 2.14.2, and/or SAS v9.4 [19]. Metabolite profile distinctions between CRC and adjacent mucosa were evaluated by matched pair *t* tests. An estimate of the false discovery rate (*q* value) was calculated to take into account the multiple comparisons that normally occur in metabolomic-based studies. A *q* value threshold of ≤0.10 was used to correct for false discovery of statistically significant compounds due to multiple hypothesis testing. Metabolites with *q* values that exceeded this threshold were discarded from further analysis. Fold difference (FD) was determined by dividing the relative abundance of the metabolite in the CRC by the relative abundance of the metabolite in the adjacent mucosa. Metabolites with *p* values of ≤0.05 with *q* values below the threshold of ≤0.10 were considered statistically significant in this study.

### Metabolic pathway networks and analysis

To visualize and analyze small molecules within relevant networks of metabolic pathways, the detected metabolites in CRC and adjacent mucosa were subjected to MetaboLync pathway analysis (MPA) software (portal.metabolon.com). Significantly altered pathways were determined by completing pathway set enrichment analysis within MPA software which was determined by the following equation:

# of significant metabolites in pathway (*k*)/total # of detected metabolites in pathway (*m*)/total # of significant metabolites (*n*)/total # of detected metabolites (*N*) or (*k*/*m*)/(*n*/*N*).

A pathway impact score greater than one indicates that the pathway contains a higher number of experimentally regulated compounds relative to the overall study in CRC than adjacent mucosa, suggesting that the pathway may be of interest to the metabolite perturbations observed. Finally, the significantly altered pathways and metabolites were visualized via a Pathway Visualizations tool using Cytoscape v 2.8.3 software [20].

## Results

### Metabolite differences between CRC and adjacent mucosa

**Fig. 1:**
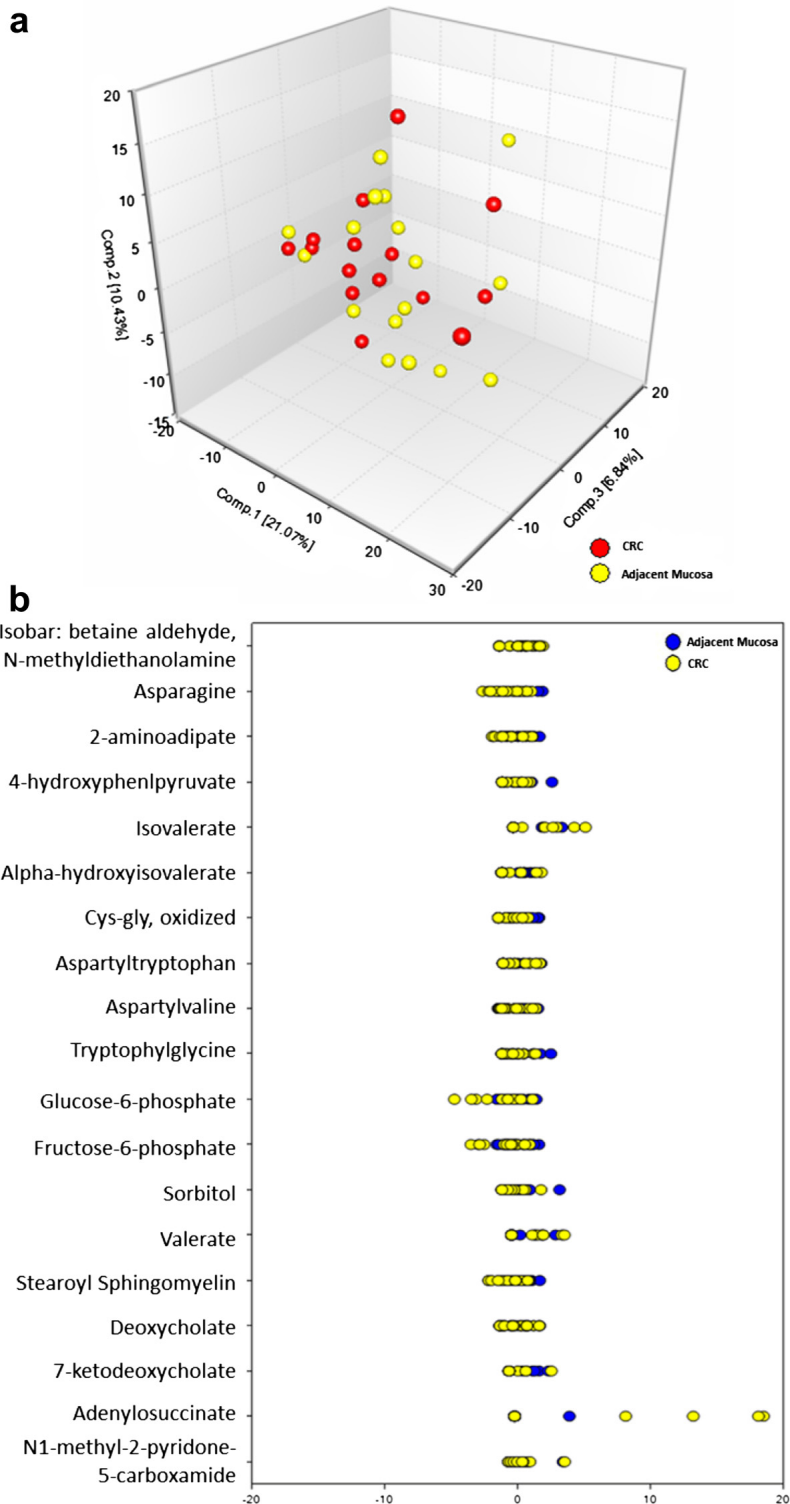
Metabolomics analysis of CRC and adjacent mucosa. **a** PCA CRC and adjacent mucosa and **b** *z*-scores for the 19 significantly different metabolites and varied expression between CRC and adjacent mucosa

Principal component analysis (PCA) of CRC and adjacent mucosa samples revealed no distinct clustering by tissue type (Fig. 1a). *z*-scores for 19 metabolites significantly different between CRC and adjacent mucosa are illustrated in Fig. 1b. Table 2 lists the metabolites and metabolic pathways with significant differences between CRC and adjacent mucosa in 17 patients (*p* ≤ 0.05). Data are presented as the mean fold difference in CRC metabolite abundance compared to adjacent mucosa for all patients. The 5 metabolites that showed higher abundance in CRC includes isobar: betaine aldehyde, N-methyldiethanolamine (2.68-fold) representing glycine, serine, and threonine metabolic pathways, adenylosuccinate (1.88-fold) representing the purine (adenine containing) metabolic pathway, isovalerate (1.45-fold) representing leucine, isoleucine, and valine metabolic pathways, valerate (1.37-fold) representing the short-chain fatty acid metabolic pathway, and N1-methyl-2-pyridone-carboxamide (1.29-fold) representing nicotinate and nicotinamide metabolic pathways. Table 2 further shows the 14 metabolites with lower abundance in CRC compared to mucosa. These include 2-aminoadipate (0.93-fold) representing the lysine metabolic pathway, stearoyl sphingomyelin (0.9-fold) representing the sphingolipid metabolic pathway, 4-hydroxyphenylpyruvate (0.88-fold) representing phenylalanine and tyrosine metabolic pathways, sorbitol (0.87-fold) representing fructose, mannose, and galactose metabolic pathways, alpha-hydroxyisovalerate (0.86-fold) representing leucine, isoleucine, and valine metabolic pathways, cys-gly, oxidized (0.84-fold) representing the glutathione metabolic pathway, trytophylglycine (0.84-fold), aspartylvaline (0.81-fold), and aspartyltryptophan (0.76-fold) representing the dipeptide metabolic pathway, deoxycholate (0.84-fold) and 7-ketodeoxycholate (0.81-fold) representing secondary bile acid metabolism, asparagine (0.81-fold) representing alanine and aspartate metabolic pathways, and glucose-6-phosphate (0.64-fold) and fructose-6-phosphate (0.75-fold) representing glycolytic, gluconeogenic, and pyruvate metabolic pathways. The information on the metabolic pathway, metabolite name, platform of detection, Kyoto Encyclopedia of Genes and Genomes (KEGG), Human Metabolome DataBase (HMDB), and PubChem identifiers for all 19 metabolites is listed in Additional file 1: Table S1.

**Table 2:** Metabolites with statistically significant differences between colorectal cancer tissue and adjacent mucosa

Metabolic pathway	Metabolite	*p* value	Fold difference (CRC/mucosa)
Glycine, serine, and threonine metabolism	Isobar: betaine aldehyde, N-methyldiethanolamine	0.015	2.68↑
Purine metabolism, adenine containing	Adenylosuccinate	0.03	1.88↑
Leucine, isoleucine, and valine metabolism	Isovalerate	0.0073	1.45↑
Short-chain fatty acid	Valerate	0.013	1.37↑
Nicotinate and nicotinamide metabolism	N1-methyl 2-pyridone-5-carboxamide	0.018	1.29↑
Lysine metabolism	2-aminoadipate	0.043	0.93↓
Sphingolipid metabolism	Stearoyl sphingomyelin	0.049	0.9↓
Phenylalanine and tyrosine metabolism	4-hydroxyphenylpyruvate	0.05	0.88↓
Fructose, mannose, and galactose metabolism	Sorbitol	0.029	0.87↓
Leucine, isoleucine, and valine metabolism	Alpha-hydroxyisovalerate	0.037	0.86↓
Glutathione metabolism	Cys-gly, oxidized	0.047	0.84↓
Dipeptide	Tryptophylglycine	0.041	0.84↓
Secondary bile acid metabolism	Deoxycholate	0.045	0.84↓
	7-ketodeoxycholate	0.04	0.81↓
Alanine and aspartate metabolism	Asparagine	0.025	0.81↓
Dipeptide	Aspartylvaline	0.035	0.81↓
	Aspartyltryptophan	0.019	0.76↓
Glycolysis, gluconeogenesis, and pyruvate metabolism	Fructose-6-phosphate	0.0082	0.75↓
	Glucose-6-phosphate (G6P)	0.0025	0.64↓

↑ designates metabolites with significantly (*p* ≤ 0.05) higher expression in CRC when compared with adjacent mucosa (metabolite ratio of ≥1.00) and ↓ designates metabolites with significantly (*p* ≤ 0.05) higher expression in adjacent mucosa when compared with CRC (metabolite ratio of <1.00)

### Metabolic network differences between CRC and adjacent mucosa

**Fig. 2:**
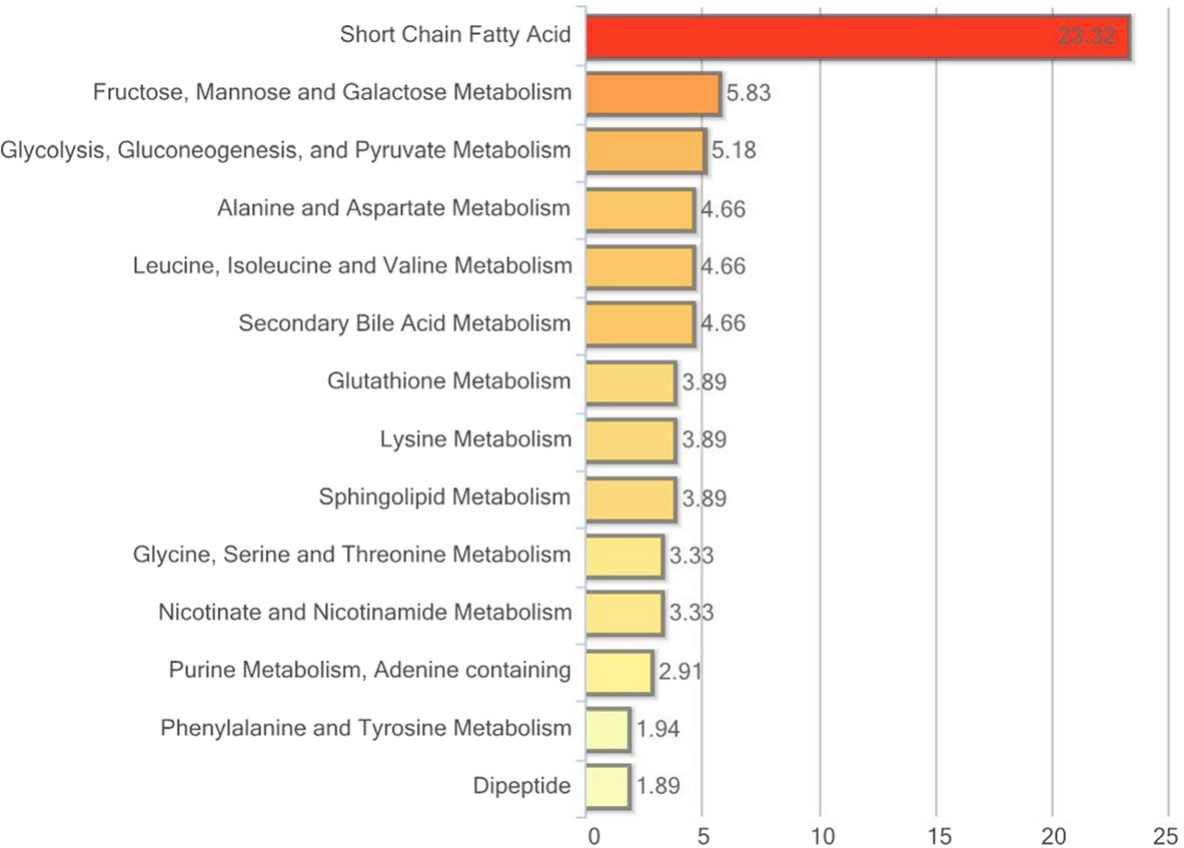
Pathway set enrichment of metabolic networks distinguished between CRC and adjacent mucosa. There were 14 pathway distinctions determined by a pathway impact score greater than 1. The pathway impact score was determined as defined in the “Methods” section

**Fig. 3:**
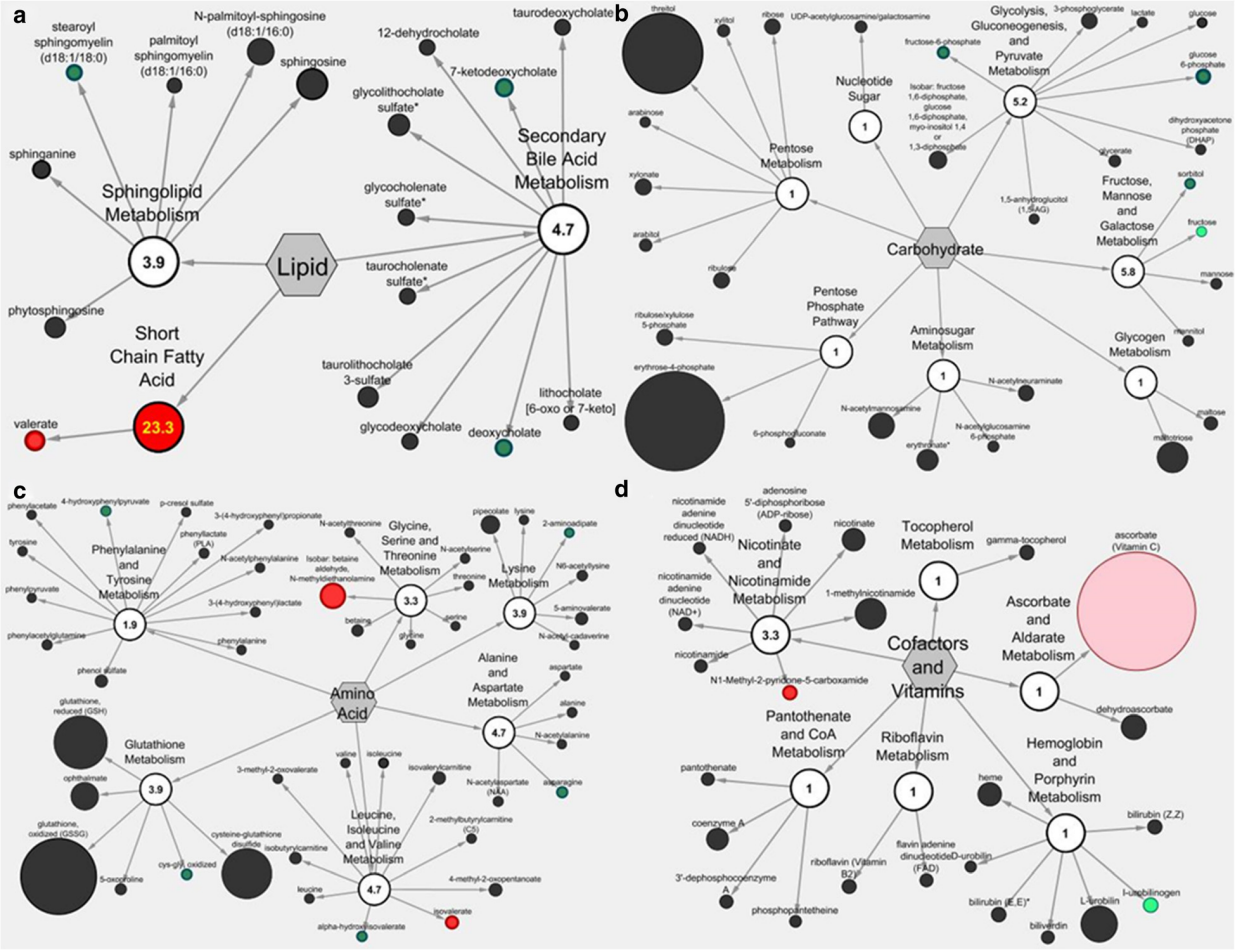
Cytoscape Pathway Classification Network view of CRC versus adjacent mucosa metabolites. Metabolites and metabolic pathways are represented by nodes with a numeric pathway impact score for (**a**) lipid (**b**) carbohydrate (**c**) amino acid and (**d**) cofactors and vitamin metabolism. Nodes are colored based on significance with green representing metabolites with lower expression in the CRC compared to adjacent mucosa. Red nodes represent metabolites and pathways with higher expression in CRC (p ≤ 0.05). Light green and light red coloring represents metabolites and pathways that showed trends towards significance (0.05 < p < 0.10)

The pathway set enrichment analysis was performed to elucidate the metabolic pathways affected by metabolite distinctions between CRC and adjacent mucosa. This analysis revealed significant perturbation of 14 metabolic networks, including but not limited to short-chain fatty acid (23.32), fructose, mannose, and galactose (5.83), and glycolytic, gluconeogenic, and pyruvate (5.18) metabolic pathways (Fig. 2). The Cytoscape Pathway Classification Network view of impacted metabolic networks for lipid, carbohydrate, amino acid, and cofactors and vitamin pathway metabolites are shown in Fig. 3. Figure 3 shows the following CRC discriminatory metabolites in their respective metabolic network. These are stearoyl sphingomyelin (sphingolipid metabolism), valerate (short-chain fatty acid metabolism), 7-ketodeoxycholate and deoxycholate (secondary bile acid metabolism), fructose-6-phosphate and glucose-6-phosphate (glycolysis, gluconeogenesis, and pyruvate metabolism), sorbitol (fructose, mannose, and galactose metabolism), 4-hydroxyphenylpruvate (phenylalanine and tyrosine metabolism), isobar: betaine aldehyde, N-methyldiethanolamine (glycine, serine, and threonine metabolism), 2-aminoadipate (lysine metabolism), asparagine (alanine and aspartate metabolism), cys-gly, oxidized (glutathione metabolism), alpha-hydroxyisovalerate and valerate (leucine, isoleucine, and valine metabolism), and N1-methyl-2-pyridone-5-carboxamide (nicotinate and nicotinamide metabolism).

### Stool metabolome of CRC patients

In this study, we detected 500 metabolites in the stool matrix of 13 CRC patients. The metabolic pathway, metabolite name, platform of detection, KEGG, HMDB, and PubChem identifiers of all detected stool metabolites can be found in Additional file 1: Table S2. This includes 102 amino acid metabolites representing alanine and aspartate, glutamate, glutathione, glycine, serine, and threonine, histidine, leucine, isoleucine, and valine, lysine, methionine, cysteine, SAM, and taurine, phenylalanine and tyrosine, polyamine, tryptophan, and urea cycle; arginine and proline metabolic pathways. It also included 27 carbohydrate metabolites representing aminosugar, disaccharides and oligosaccharides, fructose, mannose, and galactose, glycogen, glycolysis, gluconeogenesis, and pyruvate, pentose, and metabolic pathways. Twenty cofactors and vitamin metabolites representing ascorbate and aldarate, hemoglobin and porphyrin, nicotinate and nicotinamide, pantothenate and CoA, riboflavin, tetrahydrobiopterin, thiamine, tocopherol, and vitamin B6 metabolic pathways were identified. Additionally, we observed 7 energy metabolites in the stool metabolome including oxidative phosphorylation and TCA cycle intermediates. The largest portion of the CRC stool metabolome were metabolites involved in lipid metabolism representing carnitine, endocannabinoid, fatty acid, fatty alcohol, long chain, glycerolipid, inositol, ketone bodies, lysolipid, monoacylglycerol, phospholipid, primary and secondary bile acid, sphingolipid, steroid, and sterol metabolic pathways. There were 25 nucleotide metabolites representing purine and pyrimidine metabolism, 106 peptide metabolites representing dipeptide, gamma-glutamyl amino acid, and polypeptide metabolism, and 71 xenobiotics representing chemical, drug, food component/plant and xanthine metabolic pathways (Table 3).

**Table 3:** CRC stool metabolite numbers within each of the assigned metabolic pathways

Metabolic pathway	Number of metabolites
Amino acid	102
Alanine and aspartate	5
Glutamate	4
Glutathione	1
Glycine, serine, and threonine	6
Histidine	5
Leucine, isoleucine, and valine	17
Lysine	8
Methionine, cysteine, SAM, and taurine	9
Phenylalanine and tyrosine	23
Polyamine	6
Tryptophan	10
Urea cycle; arginine and proline	8
Carbohydrate	27
Aminosugar	6
Disaccharides and oligosaccharides	2
Fructose, mannose, and galactose	4
Glycogen	1
Glycolysis, gluconeogenesis, and pyruvate	3
Pentose	11
Cofactors and vitamins	20
Ascorbate and aldarate	1
Hemoglobin and porphyrin	5
Nicotinate and nicotinamide	4
Pantothenate and CoA	1
Riboflavin	2
Tetrahydrobiopterin	1
Thiamine	2
Tocopherol	3
Vitamin B6	1
Energy	7
Oxidative phosphorylation	1
TCA cycle	6
Lipid	142
Carnitine metabolism	3
Endocannabinoid	2
Fatty acid	56
Fatty alcohol, long chain	2
Glycerolipid	2
Inositol	2
Ketone bodies	1
Lysolipid	16
Monoacylglycerol	8
Phospholipid	1
Primary bile acid	6
Secondary bile acid	12
Sphingolipid	4
Steroid	9
Sterol	7
Nucleotide	25
Purine and pyrimidine	1
Purine metabolism	5
(Hypo) xanthine/inosine containing	2
Purine metabolism, adenine containing	4
Purine metabolism, guanine containing	1
Pyrimidine metabolism, cytidine containing	1
Pyrimidine metabolism, orotate containing	4
Pyrimidine metabolism, thymine containing	7
Pyrimidine metabolism, uracil containing	
Peptide	106
Dipeptide	99
Gamma-glutamyl amino acid	4
Polypeptide	3
Xenobiotics	71
Benzoate	4
Chemical	13
Drug	10
Food component/plant	33
Xanthine	11

### Metabolome overlap and comparison between CRC and stool

**Fig. 4:**
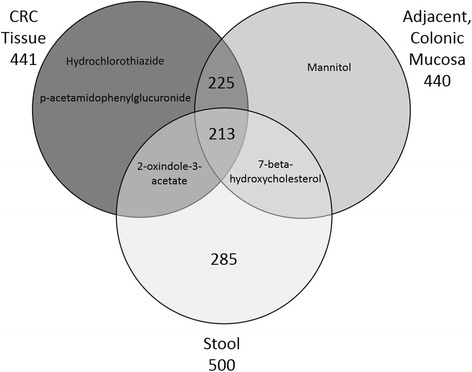
Venn diagram of the total number of metabolites detected across CRC, adjacent mucosa, and stool samples from (*n* = 17) colorectal cancer patients. Nearly all metabolite detections were shared across CRC and adjacent mucosal tissue. Two-hundred fifteen metabolites from the stool metabolome were also detected in tissue matrices

We next identified the metabolome overlap and differences across CRC, adjacent mucosa, and stool. A total of 728 small molecules were detected in CRC, adjacent mucosa, and stool collected from recently diagnosed CRC patients undergoing colonic resection. Compared to the 19 discriminatory metabolites between CRC and adjacent mucosa, only 7 of these discriminatory metabolites were also detected in the stool metabolome. These metabolites were alpha-hydroxyisovalerate, isovalerate, N1-methyl-2-pyridine-5-carboxamide, 7-ketodeoxycholate, deoxycholate, valerate, and tryptophylglycine. The Venn diagram in Fig. 4 shows the 213 metabolites that were common to all 3 sample matrices when compared to the 728 total metabolites detected. Hydrochlorothiazide and p-acetamidophenylglucuronide were identified only in CRC whereas mannitol was specific to adjacent mucosa. There were 285 unique stool metabolites, of which 214 stool metabolites overlapped in mucosa, and 214 metabolites shared between stool and CRC. 7-beta-hydroxycholesterol and 2-oxindole-3-acetate were uniquely shared between stool and CRC and stool and mucosa, respectively. Additional file 1: Figure S1 further illustrates the distinct and overlapping tissue and stool metabolites of gut microbial and host origins [21].

## Discussion

This multi-platform CRC and adjacent mucosa metabolome analysis led to the identification of 11 novel metabolites that have not previously been detected for CRC relevance in animals or humans. Adenylosuccinate, isovalerate, valerate, N1-methyl-2-pyridone-5-carboxamide, stearoyl sphingomyelin, 4-hydroxyphenylpyruvate, aspartylvaline, aspartyltryptophan, tryptophyglycine, glucose-6-phosphate, and fructose-6-phosphate merit further investigation as key small molecules in CRC pathogenesis. It is noteworthy that tryptophylglycine was detected in a fecal metabolomics study comparing CRC patients and matched healthy controls [22], whereas our report is the first to our knowledge to show tryptophylglycine metabolite levels in human tissue. One limitation of this study is the small sample size and subsequent low representation of each tumor stage and location. A strength of this pilot investigation was the capacity to obtain CRC and adjacent mucosa with stool samples from the same patient and utilize a multi-platform analytical approach that is comparable to other human datasets (GC/LC-MS) [8, 9, 23].

Our findings supported the identification of 8 metabolites that were discriminatory between CRC and adjacent mucosa in previous reports and included 2-aminoadipate, isobar: betaine aldehyde, N-methyldiethanolamine, alpha-hydroxyisovalerate, cys-gly, oxidized, deoxycholate, 7-ketodeoxycholate, sorbitol, and asparagine [9, 14, 23]. However, these results were not consistent by the relative abundance, such that the asparagine, 2-aminoadipate, alpha-hydroxyisovalerate, deoxycholate, 7-ketodeoxycholate, sorbitol and cys-gly, oxidized had higher expression in adjacent mucosa compared to CRC, whereas isobar: betaine aldehyde, N-methyldiethanolamine had higher expression in CRC than in adjacent mucosa in this study [14]. Intraindividual differences in tumor heterogeneity and analytical platforms may, in part, explain these discrepancies [11].

Two secondary bile acid metabolites (7-ketodeoxycholate and deoxycholate) showed higher expression in adjacent mucosa compared with CRC and were detectable across sample matrices (Additional file 1: Table S1). These metabolites are associated with generation of reactive oxygen and nitrogen species [24–27], altering the stability of the cell and mitochondrial membranes [25, 26], and inducing oxidative DNA damage [25, 26], mutation [25, 26], and apoptosis in CRC [25, 26]. Deoxycholic acid was shown to be diet-modifiable in the plasma metabolome [28], and had higher expression in adjacent mucosa in this study. Alternatively, the variable levels we observed between tissue matrices could be due to gut microbial metabolism. The gut mircobiota are known to convert primary bile acids to secondary bile acids, a process that may be pH-dependent [29]. Variable gut microbial populations throughout the colon and from person to person may also contribute to the differential expression of secondary bile acids observed in this study (Fig. 3a).

Two additional key small molecules of CRC pathogenesis were elucidated in this global, metabolite profiling: N1-methyl-2-pyridone-5-carboxamide and sorbitol. These small molecules are important because they were discriminatory between CRC and adjacent mucosa in this study and they have been reported in disease contexts including chronic renal failure and diabetes [30, 31]. N1-methyl 2-pyridone-5-carboxamide (N125) has been increased in high fat diet-induced obesity, which is an elevated CRC risk factor [32]. N125 was also shown as diet-modifiable in urinary metabolomes [28], supporting its possible importance as a diet-modifiable mediator of CRC pathogenesis. Additionally, excess sorbitol has been associated with diabetic-related microvascular complications and retinopathy, and its accumulation was associated with osmotic and oxidative stress damage to the endothelium [30].

Beyond specific metabolite differences, we also evaluated entire metabolic networks to interrogate their contributions to both CRC and mucosal tissue microenvironments. The perturbation of short-chain fatty acid metabolism between CRC and adjacent mucosa is noteworthy because of the breadth of short-chain fatty acid metabolites emphasized in CRC prevention and control research. Short-chain fatty acids such as butyrate, propionate, and valerate have been shown to cause growth arrest and differentiation in human colorectal cancer cells [33]. In our study, short-chain fatty acid metabolism had a pathway impact score of 23.32 and contained the discriminatory metabolite valerate. These results taken together merit continued metabolomics investigation of short-chain fatty acids in the context of CRC pathogenesis and prevention, specifically valerate [34–36]. In addition to short-chain fatty acid metabolism, our pathway analysis revealed linkages to the glycolytic/gluconeogenic pathway and CRC tumorigenesis (pathway impact score of 5.8). This metabolic pathway contained 2 discriminatory metabolites: fructose-6-phosphate and glucose-6-phosphate. Glucose-6-phosphate and fructose-6-phosphate represent metabolites/intermediates of cellular respiration that are established for enhancing cancer cell energy required for rapid proliferation [37]. In this study, we observed depleted glucose-6-phosphate and fructose-6-phosphate levels in tumor tissues relative to adjacent mucosa which implies a Warburg effect or a switch from oxidative to substrate-level phosphorylation for energy production [38]. Larger cohort investigations may be needed to establish the signatures associated with short-chain fatty acid metabolism, fructose, mannose, and galactose metabolism, glycolysis, gluconeogenesis, and pyruvate metabolism, alanine and aspartate metabolism, leucine, isoleucine, and valine metabolism, and secondary bile acid metabolism (Fig. 2). Given that the CRC metabotype has not yet been expanded to provide metabolite information specific to the colonic location affected (i.e., proximal, distal, rectum), future investigations should consider this to be another experimental parameter by which to discriminate samples from each other and for enhanced precision in biomarker discovery [8].

Of the 703 metabolites detected in this study, 29.2 % of metabolites overlap across tissue and stool matrices (Fig. 4). Colorectal tissue and stool metabolite profiles derive from several inputs including altered gene expression, oxidative stress responses, xenobiotic metabolism, and the utilization of alternative carbon sources from the gut microbiota. Findings from this pilot investigation of CRC and stool raises awareness regarding the utility of stool metabolites as relevant indicators of CRC tissue microenvironment [39, 40].

## Conclusions

This pilot, global, non-targeted metabolome study identified various metabolic alterations in both individual metabolites and metabolic pathway networks between CRC and adjacent mucosa from CRC patients. In addition, we observed a low percentage of conserved detection across tissue and stool matrices (213 shared metabolites of 728 total metabolites; 29.2 % conserved metabolites). Findings from this study reiterate the complexity of CRC biology with regard to individual phenotypes and the utility for a broad-spectrum metabolite detection platform to guide our approaches to treatment, control, and prevention of this complex malignancy.

## Abbreviations

ANOVA, analysis of variance; *APC*, adenomatous polyposis coli; *BRAF*, B-Raf proto-oncogene, serine/threonine kinase; CEA, carcinoembryonic antigen; COX, cyclooxygenase; CRC, colorectal cancer; FD, fold difference; GC/MS, gas chromatography mass spectrometry; HMDB, human metabolome database; KEGG, Kyoto Encyclopedia Genes and Genomes; *KRAS*, Kirsten rat sarcoma oncogene homolog; LC-MS/MS, liquid chromatography mass spectrometry; m/z, mass-to-charge ratio; MPA, MetaboLync pathway analysis; MSI, microsatellite instability; NSAID, non-steroidal anti-inflammatory drugs; PVH, Poudre Valley Hospital; RI/RT, retention index/retention time.

## Additional file

Additional file 1: 2016 metabolomics investigation supplemental file. Supplemental Table S1. Supplemental Table S2. Supplemental Figure S1. (DOCX 1332 kb)
