# Supplementary material for: Metabolomics and metabolic pathway networks from human colorectal cancers, adjacent mucosa, and stool
Source: Cancer Metab. 2016 Jun 6;4:11. doi: 10.1186/s40170-016-0151-y (PMC4893840; doi:10.1186/s40170-016-0151-y)
Supplement: Additional file 1: — 2016 metabolomics investigation supplemental file. Supplemental Table S1. Supplemental Table S2. Supplemental Figure S1. (DOCX 1332 kb) [file 40170_2016_151_MOESM1_ESM.docx]

**Supplemental Tables**

| **Supplementary Table S1.** Individual metabolite characteristics for all identified metabolites with statistically significance between CRC and adjacent mucosa by global profiling. | | | | | | | |
| --- | --- | --- | --- | --- | --- | --- | --- |
| Super Pathway | Sub Pathway | Metabolite | Platform | KEGG | HMDB | PubChem | Matrix Detected |
| Amino Acid | Leucine, Isoleucine and Valine Metabolism | alpha-hydroxyisovalerate | GC/MS |  | [HMDB00407](http://www.hmdb.ca/metabolites/HMDB00407) | 99823 | Stool + Tissue |
| Amino Acid | Leucine, Isoleucine and Valine Metabolism | isovalerate | LC/MS neg | [C08262](http://www.genome.jp/dbget-bin/www_bget?cpd+C08262) | [HMDB00718](http://www.hmdb.ca/metabolites/HMDB00718) | 10430 | Stool + Tissue |
| Cofactors and Vitamins | Nicotinate and Nicotinamide Metabolism | N1-Methyl-2-pyridone-5-carboxamide | LC/MS pos | [C05842](http://www.genome.jp/dbget-bin/www_bget?cpd+C05842) | [HMDB04193](http://www.hmdb.ca/metabolites/HMDB04193) | 69698 | Stool + Tissue |
| Lipid | Secondary Bile Acid Metabolism | 7-ketodeoxycholate | LC/MS neg |  | [HMDB00391](http://www.hmdb.ca/metabolites/HMDB00391) | 188292 | Stool + Tissue |
| Lipid | Secondary Bile Acid Metabolism | deoxycholate | GC/MS | [C04483](http://www.genome.jp/dbget-bin/www_bget?cpd+C04483) | [HMDB00626](http://www.hmdb.ca/metabolites/HMDB00626) | 222528 | Stool + Tissue |
| Lipid | Short Chain Fatty Acid | valerate | LC/MS neg | [C00803](http://www.genome.jp/dbget-bin/www_bget?cpd+C00803) | [HMDB00892](http://www.hmdb.ca/metabolites/HMDB00892) | 7991 | Stool + Tissue |
| Peptide | Dipeptide | tryptophylglycine | LC/MS pos |  |  | 263471 | Stool + Tissue |
| Amino Acid | Alanine and Aspartate Metabolism | asparagine | GC/MS | [C00152](http://www.genome.jp/dbget-bin/www_bget?cpd+C00152) | [HMDB00168](http://www.hmdb.ca/metabolites/HMDB00168) | 6267 | Tissue |
| Amino Acid | Glutathione Metabolism | cys-gly, oxidized | GC/MS |  |  | 333293 | Tissue |
| Amino Acid | Glycine, Serine and Threonine Metabolism | Isobar: betaine aldehyde, N-methyldiethanolamine | LC/MS pos |  |  | 249 | Tissue |
| Amino Acid | Lysine Metabolism | 2-aminoadipate | GC/MS | [C00956](http://www.genome.jp/dbget-bin/www_bget?cpd+C00956) | [HMDB00510](http://www.hmdb.ca/metabolites/HMDB00510) | 469 | Tissue |
| Amino Acid | Phenylalanine and Tyrosine Metabolism | 4-hydroxyphenylpyruvate | LC/MS neg | [C01179](http://www.genome.jp/dbget-bin/www_bget?cpd+C01179) | [HMDB00707](http://www.hmdb.ca/metabolites/HMDB00707) | 979 | Tissue |
| Carbohydrate | Fructose, Mannose and Galactose Metabolism | sorbitol | GC/MS | [C00794](http://www.genome.jp/dbget-bin/www_bget?cpd+C00794) | [HMDB00247](http://www.hmdb.ca/metabolites/HMDB00247) | 5780 | Tissue |
| Carbohydrate | Glycolysis, Gluconeogenesis, and Pyruvate Metabolism | fructose-6-phosphate | GC/MS | [C05345](http://www.genome.jp/dbget-bin/www_bget?cpd+C05345) | [HMDB00124](http://www.hmdb.ca/metabolites/HMDB00124) | 69507 | Tissue |
| Carbohydrate | Glycolysis, Gluconeogenesis, and Pyruvate Metabolism | glucose-6-phosphate (G6P) | GC/MS | [C00668](http://www.genome.jp/dbget-bin/www_bget?cpd+C00668) | [HMDB01401](http://www.hmdb.ca/metabolites/HMDB01401) | 5958 | Tissue |
| Lipid | Sphingolipid Metabolism | stearoyl sphingomyelin | GC/MS | [C00550](http://www.genome.jp/dbget-bin/www_bget?cpd+C00550) | [HMDB01348](http://www.hmdb.ca/metabolites/HMDB01348) | 6453725 | Tissue |
| Nucleotide | Purine Metabolism, Adenine containing | adenylosuccinate | LC/MS neg | [C03794](http://www.genome.jp/dbget-bin/www_bget?cpd+C03794) | [HMDB00536](http://www.hmdb.ca/metabolites/HMDB00536) | 195 | Tissue |
| Peptide | Dipeptide | aspartyltryptophan | LC/MS pos |  |  | 9948926 | Tissue |
| Peptide | Dipeptide | aspartylvaline | LC/MS pos |  |  | 4991131 | Tissue |
|  | | | | | | | |

| **Supplementary Table S2.** Individual metabolite characteristics for all identified metabolites in the stool matrix. | | | | | | |
| --- | --- | --- | --- | --- | --- | --- |
| Sub Pathway | Metabolite | Platform | KEGG | HMDB | PubChem |  |
| Alanine and Aspartate Metabolism | alanine | GC/MS | [C00041](http://www.genome.jp/dbget-bin/www_bget?cpd+C00041) | [HMDB00161](http://www.hmdb.ca/metabolites/HMDB00161) | 5950 |  |
|  | aspartate | GC/MS | [C00049](http://www.genome.jp/dbget-bin/www_bget?cpd+C00049) | [HMDB00191](http://www.hmdb.ca/metabolites/HMDB00191) | 5960 |  |
|  | N-acetylalanine | LC/MS Neg | [C02847](http://www.genome.jp/dbget-bin/www_bget?cpd+C02847) | [HMDB00766](http://www.hmdb.ca/metabolites/HMDB00766) | 88064 |  |
|  | N-acetylaspartate (NAA) | GC/MS | [C01042](http://www.genome.jp/dbget-bin/www_bget?cpd+C01042) | [HMDB00812](http://www.hmdb.ca/metabolites/HMDB00812) | 65065 |  |
|  | N-propionylalanine | LC/MS Pos |  |  | 321155 |  |
| Glutamate Metabolism | gamma-aminobutyrate (GABA) | GC/MS | [C00334](http://www.genome.jp/dbget-bin/www_bget?cpd+C00334) | [HMDB00112](http://www.hmdb.ca/metabolites/HMDB00112) | 119 |  |
|  | glutamate | LC/MS Pos | [C00025](http://www.genome.jp/dbget-bin/www_bget?cpd+C00025) | [HMDB00148](http://www.hmdb.ca/metabolites/HMDB00148) | 611 |  |
|  | glutamine | LC/MS Pos | [C00064](http://www.genome.jp/dbget-bin/www_bget?cpd+C00064) | [HMDB00641](http://www.hmdb.ca/metabolites/HMDB00641) | 5961 |  |
|  | N-acetylglutamate | LC/MS Neg | [C00624](http://www.genome.jp/dbget-bin/www_bget?cpd+C00624) | [HMDB01138](http://www.hmdb.ca/metabolites/HMDB01138) | 70914 |  |
| Glutathione Metabolism | 5-oxoproline | LC/MS Neg | [C01879](http://www.genome.jp/dbget-bin/www_bget?cpd+C01879) | [HMDB00267](http://www.hmdb.ca/metabolites/HMDB00267) | 7405 |  |
| Glycine, Serine and Threonine Metabolism | allo-threonine | GC/MS | [C05519](http://www.genome.jp/dbget-bin/www_bget?cpd+C05519) | [HMDB04041](http://www.hmdb.ca/metabolites/HMDB04041) | 99289 |  |
|  | glycine | GC/MS | [C00037](http://www.genome.jp/dbget-bin/www_bget?cpd+C00037) | [HMDB00123](http://www.hmdb.ca/metabolites/HMDB00123) | 750 |  |
|  | homoserine | GC/MS | [C00263](http://www.genome.jp/dbget-bin/www_bget?cpd+C00263) | [HMDB00719](http://www.hmdb.ca/metabolites/HMDB00719) | 12647 |  |
|  | N-acetylthreonine | LC/MS Neg | [C01118](http://www.genome.jp/dbget-bin/www_bget?cpd+C01118) |  | 152204 |  |
|  | serine | GC/MS | [C00065](http://www.genome.jp/dbget-bin/www_bget?cpd+C00065) | [HMDB00187](http://www.hmdb.ca/metabolites/HMDB00187) | 5951 |  |
|  | threonine | LC/MS Pos | [C00188](http://www.genome.jp/dbget-bin/www_bget?cpd+C00188) | [HMDB00167](http://www.hmdb.ca/metabolites/HMDB00167) | 6288 |  |
| Histidine Metabolism | formiminoglutamate | LC/MS Pos | [C00439](http://www.genome.jp/dbget-bin/www_bget?cpd+C00439) |  | 439233 |  |
|  | histamine | GC/MS | [C00388](http://www.genome.jp/dbget-bin/www_bget?cpd+C00388) | [HMDB00870](http://www.hmdb.ca/metabolites/HMDB00870) | 774 |  |
|  | histidine | LC/MS Neg | [C00135](http://www.genome.jp/dbget-bin/www_bget?cpd+C00135) | [HMDB00177](http://www.hmdb.ca/metabolites/HMDB00177) | 6274 |  |
|  | imidazole propionate | LC/MS Pos |  | [HMDB02271](http://www.hmdb.ca/metabolites/HMDB02271) | 70630 |  |
|  | trans-urocanate | LC/MS Pos | [C00785](http://www.genome.jp/dbget-bin/www_bget?cpd+C00785) | [HMDB00301](http://www.hmdb.ca/metabolites/HMDB00301) | 736715 |  |
| Leucine, Isoleucine and Valine Metabolism | 2-hydroxy-3-methylvalerate | LC/MS Neg |  | [HMDB00317](http://www.hmdb.ca/metabolites/HMDB00317) | 164623 |  |
|  | 3-methyl-2-oxobutyrate | LC/MS Neg | [C00141](http://www.genome.jp/dbget-bin/www_bget?cpd+C00141) | [HMDB00019](http://www.hmdb.ca/metabolites/HMDB00019) | 49 |  |
|  | 3-methyl-2-oxovalerate | LC/MS Neg | [C00671](http://www.genome.jp/dbget-bin/www_bget?cpd+C00671) | [HMDB03736](http://www.hmdb.ca/metabolites/HMDB03736) | 47 |  |
|  | 4-methyl-2-oxopentanoate | LC/MS Neg | [C00233](http://www.genome.jp/dbget-bin/www_bget?cpd+C00233) | [HMDB00695](http://www.hmdb.ca/metabolites/HMDB00695) | 70 |  |
|  | allo-isoleucine | GC/MS |  |  | 6950182;99288 |  |
|  | alpha-hydroxyisocaproate | LC/MS Neg | [C03264](http://www.genome.jp/dbget-bin/www_bget?cpd+C03264) | [HMDB00746](http://www.hmdb.ca/metabolites/HMDB00746) | 83697 |  |
|  | alpha-hydroxyisovalerate | GC/MS |  | [HMDB00407](http://www.hmdb.ca/metabolites/HMDB00407) | 99823 |  |
|  | beta-hydroxyisovalerate | GC/MS |  | [HMDB00754](http://www.hmdb.ca/metabolites/HMDB00754) | 69362 |  |
|  | isoleucine | LC/MS Pos | [C00407](http://www.genome.jp/dbget-bin/www_bget?cpd+C00407) | [HMDB00172](http://www.hmdb.ca/metabolites/HMDB00172) | 6306 |  |
|  | isovalerate | LC/MS Neg | [C08262](http://www.genome.jp/dbget-bin/www_bget?cpd+C08262) | [HMDB00718](http://www.hmdb.ca/metabolites/HMDB00718) | 10430 |  |
|  | leucine | LC/MS Pos | [C00123](http://www.genome.jp/dbget-bin/www_bget?cpd+C00123) | [HMDB00687](http://www.hmdb.ca/metabolites/HMDB00687) | 6106 |  |
|  | methylsuccinate | GC/MS |  | [HMDB01844](http://www.hmdb.ca/metabolites/HMDB01844) | 10349 |  |
|  | N-acetylisoleucine | LC/MS Pos |  |  | 2802421 |  |
|  | N-acetylleucine | LC/MS Pos | [C02710](http://www.genome.jp/dbget-bin/www_bget?cpd+C02710) | [HMDB11756](http://www.hmdb.ca/metabolites/HMDB11756) | 70912 |  |
|  | N-acetylvaline | LC/MS Pos |  | [HMDB11757](http://www.hmdb.ca/metabolites/HMDB11757) | 66789 |  |
|  | norleucine | GC/MS | [C01933](http://www.genome.jp/dbget-bin/www_bget?cpd+C01933) | [HMDB01645](http://www.hmdb.ca/metabolites/HMDB01645) | 21236 |  |
|  | valine | LC/MS Pos | [C00183](http://www.genome.jp/dbget-bin/www_bget?cpd+C00183) | [HMDB00883](http://www.hmdb.ca/metabolites/HMDB00883) | 6287 |  |
| Lysine Metabolism | 5-aminovalerate | GC/MS | [C00431](http://www.genome.jp/dbget-bin/www_bget?cpd+C00431) | [HMDB03355](http://www.hmdb.ca/metabolites/HMDB03355) | 138 |  |
|  | cadaverine | GC/MS | [C01672](http://www.genome.jp/dbget-bin/www_bget?cpd+C01672) | [HMDB02322](http://www.hmdb.ca/metabolites/HMDB02322) | 273 |  |
|  | glutarate (pentanedioate) | GC/MS | [C00489](http://www.genome.jp/dbget-bin/www_bget?cpd+C00489) | [HMDB00661](http://www.hmdb.ca/metabolites/HMDB00661) | 743 |  |
|  | lysine | GC/MS | [C00047](http://www.genome.jp/dbget-bin/www_bget?cpd+C00047) | [HMDB00182](http://www.hmdb.ca/metabolites/HMDB00182) | 5962 |  |
|  | N2-acetyllysine | LC/MS Pos | [C12989](http://www.genome.jp/dbget-bin/www_bget?cpd+C12989) | [HMDB00446](http://www.hmdb.ca/metabolites/HMDB00446) | 92907 |  |
|  | N6-acetyllysine | LC/MS Pos | [C02727](http://www.genome.jp/dbget-bin/www_bget?cpd+C02727) | [HMDB00206](http://www.hmdb.ca/metabolites/HMDB00206) | 92832 |  |
|  | N-acetyl-cadaverine | LC/MS Pos |  |  |  |  |
|  | pipecolate | LC/MS Pos | [C00408](http://www.genome.jp/dbget-bin/www_bget?cpd+C00408) | [HMDB00070](http://www.hmdb.ca/metabolites/HMDB00070) | 849 |  |
| Methionine, Cysteine, SAM and Taurine Metabolism | 2-aminobutyrate | GC/MS | [C02261](http://www.genome.jp/dbget-bin/www_bget?cpd+C02261) | [HMDB00650](http://www.hmdb.ca/metabolites/HMDB00650) | 439691 |  |
|  | 2-hydroxybutyrate (AHB) | GC/MS | [C05984](http://www.genome.jp/dbget-bin/www_bget?cpd+C05984) | [HMDB00008](http://www.hmdb.ca/metabolites/HMDB00008) | 440864 |  |
|  | 3-methylthiopropionate | LC/MS Neg | [C08276](http://www.genome.jp/dbget-bin/www_bget?cpd+C08276) | [HMDB01527](http://www.hmdb.ca/metabolites/HMDB01527) | 563 |  |
|  | cysteine | GC/MS | [C00097](http://www.genome.jp/dbget-bin/www_bget?cpd+C00097) | [HMDB00574](http://www.hmdb.ca/metabolites/HMDB00574) | 5862 |  |
|  | methionine | LC/MS Pos | [C00073](http://www.genome.jp/dbget-bin/www_bget?cpd+C00073) | [HMDB00696](http://www.hmdb.ca/metabolites/HMDB00696) | 6137 |  |
|  | methionine sulfoxide | LC/MS Pos | [C02989](http://www.genome.jp/dbget-bin/www_bget?cpd+C02989) | [HMDB02005](http://www.hmdb.ca/metabolites/HMDB02005) | 158980 |  |
|  | N-acetylmethionine | LC/MS Neg | [C02712](http://www.genome.jp/dbget-bin/www_bget?cpd+C02712) | [HMDB11745](http://www.hmdb.ca/metabolites/HMDB11745) | 448580 |  |
|  | N-acetylmethionine sulfoxide | LC/MS Neg |  |  | 193368 |  |
|  | taurine | GC/MS | [C00245](http://www.genome.jp/dbget-bin/www_bget?cpd+C00245) | [HMDB00251](http://www.hmdb.ca/metabolites/HMDB00251) | 1123 |  |
| Phenylalanine and Tyrosine Metabolism | (R)-salsolinol | GC/MS | [C09642](http://www.genome.jp/dbget-bin/www_bget?cpd+C09642) | [HMDB05199](http://www.hmdb.ca/metabolites/HMDB05199) | 54456 |  |
|  | 2-pentanamido-3-phenylpropanoic acid | LC/MS Neg |  |  |  |  |
|  | 3-(3-hydroxyphenyl)propionate | GC/MS | [C11457](http://www.genome.jp/dbget-bin/www_bget?cpd+C11457) | [HMDB00375](http://www.hmdb.ca/metabolites/HMDB00375) | 91 |  |
|  | 3-(4-hydroxyphenyl)lactate | LC/MS Neg | [C03672](http://www.genome.jp/dbget-bin/www_bget?cpd+C03672) | [HMDB00755](http://www.hmdb.ca/metabolites/HMDB00755) | 9378 |  |
|  | 3-(4-hydroxyphenyl)propionate | GC/MS | [C01744](http://www.genome.jp/dbget-bin/www_bget?cpd+C01744) | [HMDB02199](http://www.hmdb.ca/metabolites/HMDB02199) | 10394 |  |
|  | 3-[3-(sulfooxy)phenyl]propanoic acid | LC/MS Neg |  |  | 187488 |  |
|  | 3-hydroxyphenylacetate | GC/MS | [C05593](http://www.genome.jp/dbget-bin/www_bget?cpd+C05593) | [HMDB00440](http://www.hmdb.ca/metabolites/HMDB00440) | 12122 |  |
|  | 3-phenylpropionate (hydrocinnamate) | LC/MS Neg | [C05629](http://www.genome.jp/dbget-bin/www_bget?cpd+C05629) | [HMDB00764](http://www.hmdb.ca/metabolites/HMDB00764) | 107 |  |
|  | 4-hydroxyphenylacetate | GC/MS | [C00642](http://www.genome.jp/dbget-bin/www_bget?cpd+C00642) | [HMDB00020](http://www.hmdb.ca/metabolites/HMDB00020) | 127 |  |
|  | cis-4-hydroxycyclohexylacetic acid | GC/MS |  | [HMDB00451](http://www.hmdb.ca/metabolites/HMDB00451) |  |  |
|  | dihydrocaffeate | LC/MS Neg | [C10447](http://www.genome.jp/dbget-bin/www_bget?cpd+C10447) | [HMDB00423](http://www.hmdb.ca/metabolites/HMDB00423) | 348154 |  |
|  | gentisate | GC/MS | [C00628](http://www.genome.jp/dbget-bin/www_bget?cpd+C00628) | [HMDB00152](http://www.hmdb.ca/metabolites/HMDB00152) | 3469 |  |
|  | N-acetylphenylalanine | LC/MS Neg | [C03519](http://www.genome.jp/dbget-bin/www_bget?cpd+C03519) | [HMDB00512](http://www.hmdb.ca/metabolites/HMDB00512) | 74839 |  |
|  | N-acetyltyrosine | LC/MS Neg |  | [HMDB00866](http://www.hmdb.ca/metabolites/HMDB00866) | 68310 |  |
|  | p-cresol | LC/MS Neg | [C01468](http://www.genome.jp/dbget-bin/www_bget?cpd+C01468) | [HMDB01858](http://www.hmdb.ca/metabolites/HMDB01858) | 2879 |  |
|  | p-cresol sulfate | LC/MS Neg | [C01468](http://www.genome.jp/dbget-bin/www_bget?cpd+C01468) | [HMDB11635](http://www.hmdb.ca/metabolites/HMDB11635) | 4615423 |  |
|  | phenethylamine (isobar with 1-phenylethanamine) | LC/MS Pos | [C02455](http://www.genome.jp/dbget-bin/www_bget?cpd+C02455) | [HMDB02017](http://www.hmdb.ca/metabolites/HMDB02017) | 7408 |  |
|  | phenol sulfate | LC/MS Neg | [C02180](http://www.genome.jp/dbget-bin/www_bget?cpd+C02180) | [HMDB60015](http://www.hmdb.ca/metabolites/HMDB60015) | 74426 |  |
|  | phenylacetate | LC/MS Neg | [C07086](http://www.genome.jp/dbget-bin/www_bget?cpd+C07086) | [HMDB00209](http://www.hmdb.ca/metabolites/HMDB00209) | 999 |  |
|  | phenylalanine | LC/MS Pos | [C00079](http://www.genome.jp/dbget-bin/www_bget?cpd+C00079) | [HMDB00159](http://www.hmdb.ca/metabolites/HMDB00159) | 6140 |  |
|  | phenyllactate (PLA) | LC/MS Neg | [C05607](http://www.genome.jp/dbget-bin/www_bget?cpd+C05607) | [HMDB00779](http://www.hmdb.ca/metabolites/HMDB00779) | 3848 |  |
|  | tyramine | LC/MS Pos | [C00483](http://www.genome.jp/dbget-bin/www_bget?cpd+C00483) | [HMDB00306](http://www.hmdb.ca/metabolites/HMDB00306) | 5610 |  |
|  | tyrosine | LC/MS Pos | [C00082](http://www.genome.jp/dbget-bin/www_bget?cpd+C00082) | [HMDB00158](http://www.hmdb.ca/metabolites/HMDB00158) | 6057 |  |
| Polyamine Metabolism | 1,3-diaminopropane | GC/MS | [C00986](http://www.genome.jp/dbget-bin/www_bget?cpd+C00986) | [HMDB00002](http://www.hmdb.ca/metabolites/HMDB00002) | 428 |  |
|  | 4-acetamidobutanoate | LC/MS Pos | [C02946](http://www.genome.jp/dbget-bin/www_bget?cpd+C02946) | [HMDB03681](http://www.hmdb.ca/metabolites/HMDB03681) | 18189 |  |
|  | agmatine | GC/MS | [C00179](http://www.genome.jp/dbget-bin/www_bget?cpd+C00179) | [HMDB01432](http://www.hmdb.ca/metabolites/HMDB01432) | 199 |  |
|  | N-acetylputrescine | LC/MS Pos | [C02714](http://www.genome.jp/dbget-bin/www_bget?cpd+C02714) | [HMDB02064](http://www.hmdb.ca/metabolites/HMDB02064) | 122356 |  |
|  | putrescine | GC/MS | [C00134](http://www.genome.jp/dbget-bin/www_bget?cpd+C00134) | [HMDB01414](http://www.hmdb.ca/metabolites/HMDB01414) | 1045 |  |
|  | spermidine | LC/MS Pos | [C00315](http://www.genome.jp/dbget-bin/www_bget?cpd+C00315) | [HMDB01257](http://www.hmdb.ca/metabolites/HMDB01257) | 1102 |  |
| Tryptophan Metabolism | indole-3-carboxylic acid | LC/MS Neg | [C19837](http://www.genome.jp/dbget-bin/www_bget?cpd+C19837) | [HMDB03320](http://www.hmdb.ca/metabolites/HMDB03320) | 69867 |  |
|  | indoleacetate | GC/MS | [C00954](http://www.genome.jp/dbget-bin/www_bget?cpd+C00954) | [HMDB00197](http://www.hmdb.ca/metabolites/HMDB00197) | 802 |  |
|  | indolelactate | GC/MS | [C02043](http://www.genome.jp/dbget-bin/www_bget?cpd+C02043) | [HMDB00671](http://www.hmdb.ca/metabolites/HMDB00671) | 92904 |  |
|  | indolepropionate | LC/MS Neg |  | [HMDB02302](http://www.hmdb.ca/metabolites/HMDB02302) | 3744 |  |
|  | N-acetyltryptophan | LC/MS Neg | [C03137](http://www.genome.jp/dbget-bin/www_bget?cpd+C03137) | [HMDB13713](http://www.hmdb.ca/metabolites/HMDB13713) | 700653 |  |
|  | serotonin (5HT) | LC/MS Pos | [C00780](http://www.genome.jp/dbget-bin/www_bget?cpd+C00780) | [HMDB00259](http://www.hmdb.ca/metabolites/HMDB00259) | 5202 |  |
|  | skatol | GC/MS | [C08313](http://www.genome.jp/dbget-bin/www_bget?cpd+C08313) | [HMDB00466](http://www.hmdb.ca/metabolites/HMDB00466) | 6736 |  |
|  | tryptamine | LC/MS Pos | [C00398](http://www.genome.jp/dbget-bin/www_bget?cpd+C00398) | [HMDB00303](http://www.hmdb.ca/metabolites/HMDB00303) | 1150 |  |
|  | tryptophan | LC/MS Pos | [C00078](http://www.genome.jp/dbget-bin/www_bget?cpd+C00078) | [HMDB00929](http://www.hmdb.ca/metabolites/HMDB00929) | 6305 |  |
|  | tryptophan betaine | LC/MS Pos | [C09213](http://www.genome.jp/dbget-bin/www_bget?cpd+C09213) | [HMDB61115](http://www.hmdb.ca/metabolites/HMDB61115) | 442106 |  |
| Urea cycle; Arginine and Proline Metabolism | arginine | GC/MS | [C00062](http://www.genome.jp/dbget-bin/www_bget?cpd+C00062) | [HMDB00517](http://www.hmdb.ca/metabolites/HMDB00517) | 232 |  |
|  | citrulline | LC/MS Pos | [C00327](http://www.genome.jp/dbget-bin/www_bget?cpd+C00327) | [HMDB00904](http://www.hmdb.ca/metabolites/HMDB00904) | 9750 |  |
|  | N-acetylproline | LC/MS Pos |  |  | 322640 |  |
|  | N-methyl proline | GC/MS |  |  | 557 |  |
|  | norvaline | GC/MS | [C01826](http://www.genome.jp/dbget-bin/www_bget?cpd+C01826) | [HMDB13716](http://www.hmdb.ca/metabolites/HMDB13716) | 824 |  |
|  | ornithine | GC/MS | [C00077](http://www.genome.jp/dbget-bin/www_bget?cpd+C00077) | [HMDB03374](http://www.hmdb.ca/metabolites/HMDB03374) | 6262 |  |
|  | proline | LC/MS Pos | [C00148](http://www.genome.jp/dbget-bin/www_bget?cpd+C00148) | [HMDB00162](http://www.hmdb.ca/metabolites/HMDB00162) | 145742 |  |
|  | trans-4-hydroxyproline | GC/MS | [C01157](http://www.genome.jp/dbget-bin/www_bget?cpd+C01157) | [HMDB00725](http://www.hmdb.ca/metabolites/HMDB00725) | 5810 |  |
| Aminosugar Metabolism | erythronate* | GC/MS |  | [HMDB00613](http://www.hmdb.ca/metabolites/HMDB00613) | 2781043 |  |
|  | glucosamine | GC/MS | [C00329](http://www.genome.jp/dbget-bin/www_bget?cpd+C00329) | [HMDB01514](http://www.hmdb.ca/metabolites/HMDB01514) | 441477 |  |
|  | N-acetylgalactosamine | GC/MS | [C01074](http://www.genome.jp/dbget-bin/www_bget?cpd+C01074) | [HMDB00212](http://www.hmdb.ca/metabolites/HMDB00212) | 35717 |  |
|  | N-acetylglucosamine | GC/MS | [C00140](http://www.genome.jp/dbget-bin/www_bget?cpd+C00140) | [HMDB00215](http://www.hmdb.ca/metabolites/HMDB00215) | 24139 |  |
|  | N-acetylmuramate | LC/MS Neg | [C02713](http://www.genome.jp/dbget-bin/www_bget?cpd+C02713) | [HMDB60493](http://www.hmdb.ca/metabolites/HMDB60493) | 5462244 |  |
|  | N-acetylneuraminate | GC/MS | [C00270](http://www.genome.jp/dbget-bin/www_bget?cpd+C00270) | [HMDB00230](http://www.hmdb.ca/metabolites/HMDB00230) | 439197 |  |
| Disaccharides and Oligosaccharides | raffinose | LC/MS Neg | [C00492](http://www.genome.jp/dbget-bin/www_bget?cpd+C00492) | [HMDB03213](http://www.hmdb.ca/metabolites/HMDB03213) | 10542 |  |
|  | sucrose | LC/MS Neg | [C00089](http://www.genome.jp/dbget-bin/www_bget?cpd+C00089) | [HMDB00258](http://www.hmdb.ca/metabolites/HMDB00258) | 5988 |  |
| Fructose, Mannose and Galactose Metabolism | fructose | GC/MS | [C00095](http://www.genome.jp/dbget-bin/www_bget?cpd+C00095) | [HMDB00660](http://www.hmdb.ca/metabolites/HMDB00660) | 5984 |  |
|  | galactose | GC/MS | [C01582](http://www.genome.jp/dbget-bin/www_bget?cpd+C01582) | [HMDB00143](http://www.hmdb.ca/metabolites/HMDB00143) | 3037556 |  |
|  | mannose | GC/MS | [C00159](http://www.genome.jp/dbget-bin/www_bget?cpd+C00159) | [HMDB00169](http://www.hmdb.ca/metabolites/HMDB00169) | 18950 |  |
|  | rhamnose | GC/MS | [C00507](http://www.genome.jp/dbget-bin/www_bget?cpd+C00507) | [HMDB00849](http://www.hmdb.ca/metabolites/HMDB00849) | 25310 |  |
| Glycogen Metabolism | maltose | GC/MS | [C00208](http://www.genome.jp/dbget-bin/www_bget?cpd+C00208) | [HMDB00163](http://www.hmdb.ca/metabolites/HMDB00163) | 10991489 |  |
| Glycolysis, Gluconeogenesis, and Pyruvate Metabolism | glucose | GC/MS | [C00031](http://www.genome.jp/dbget-bin/www_bget?cpd+C00031) | [HMDB00122](http://www.hmdb.ca/metabolites/HMDB00122) | 79025 |  |
|  | glycerate | GC/MS | [C00258](http://www.genome.jp/dbget-bin/www_bget?cpd+C00258) | [HMDB00139](http://www.hmdb.ca/metabolites/HMDB00139) | 752 |  |
|  | lactate | GC/MS | [C00186](http://www.genome.jp/dbget-bin/www_bget?cpd+C00186) | [HMDB00190](http://www.hmdb.ca/metabolites/HMDB00190) | 612 |  |
| Pentose Metabolism | 2-deoxyribose | GC/MS | [C01801](http://www.genome.jp/dbget-bin/www_bget?cpd+C01801) | [HMDB03224](http://www.hmdb.ca/metabolites/HMDB03224) | 22833604 |  |
|  | arabinose | GC/MS | [C00216](http://www.genome.jp/dbget-bin/www_bget?cpd+C00216) | [HMDB00646](http://www.hmdb.ca/metabolites/HMDB00646) | 66308 |  |
|  | arabitol | GC/MS | [C01904](http://www.genome.jp/dbget-bin/www_bget?cpd+C01904) | [HMDB01851](http://www.hmdb.ca/metabolites/HMDB01851) | 94154 |  |
|  | fucose | GC/MS | [C01018](http://www.genome.jp/dbget-bin/www_bget?cpd+C01018) | [HMDB00174](http://www.hmdb.ca/metabolites/HMDB00174) | 19466 |  |
|  | ribitol | GC/MS | [C00474](http://www.genome.jp/dbget-bin/www_bget?cpd+C00474) | [HMDB00508](http://www.hmdb.ca/metabolites/HMDB00508) | 6912 |  |
|  | ribose | GC/MS | [C00121](http://www.genome.jp/dbget-bin/www_bget?cpd+C00121) | [HMDB00283](http://www.hmdb.ca/metabolites/HMDB00283) | 5779 |  |
|  | ribulose | GC/MS | [C00309](http://www.genome.jp/dbget-bin/www_bget?cpd+C00309) | [HMDB00621](http://www.hmdb.ca/metabolites/HMDB00621) | 151261 |  |
|  | threitol | GC/MS | [C16884](http://www.genome.jp/dbget-bin/www_bget?cpd+C16884) | [HMDB04136](http://www.hmdb.ca/metabolites/HMDB04136) | 169019 |  |
|  | xylitol | GC/MS | [C00379](http://www.genome.jp/dbget-bin/www_bget?cpd+C00379) | [HMDB02917](http://www.hmdb.ca/metabolites/HMDB02917) | 6912 |  |
|  | xylonate | GC/MS | [C05411](http://www.genome.jp/dbget-bin/www_bget?cpd+C05411) | [HMDB60256](http://www.hmdb.ca/metabolites/HMDB60256) | 6602431 |  |
|  | xylose | GC/MS | [C00181](http://www.genome.jp/dbget-bin/www_bget?cpd+C00181) | [HMDB00098](http://www.hmdb.ca/metabolites/HMDB00098) | 135191 |  |
| Ascorbate and Aldarate Metabolism | arabonate | GC/MS | [C00878](http://www.genome.jp/dbget-bin/www_bget?cpd+C00878) | [HMDB00539](http://www.hmdb.ca/metabolites/HMDB00539) | 122045 |  |
| Hemoglobin and Porphyrin Metabolism | bilirubin (Z,Z) | LC/MS Neg | [C00486](http://www.genome.jp/dbget-bin/www_bget?cpd+C00486) | [HMDB00054](http://www.hmdb.ca/metabolites/HMDB00054) | 5280352 |  |
|  | biliverdin | LC/MS Neg | [C00500](http://www.genome.jp/dbget-bin/www_bget?cpd+C00500) | [HMDB01008](http://www.hmdb.ca/metabolites/HMDB01008) | 5353439 |  |
|  | D-urobilin | LC/MS Pos | [C05795](http://www.genome.jp/dbget-bin/www_bget?cpd+C05795) | [HMDB04161](http://www.hmdb.ca/metabolites/HMDB04161) | 6276321 |  |
|  | I-urobilinogen | LC/MS Neg | [C05790](http://www.genome.jp/dbget-bin/www_bget?cpd+C05790) | [HMDB04157](http://www.hmdb.ca/metabolites/HMDB04157) | 26818 |  |
|  | L-urobilin | LC/MS Pos | [C05793](http://www.genome.jp/dbget-bin/www_bget?cpd+C05793) | [HMDB04159](http://www.hmdb.ca/metabolites/HMDB04159) | 5280818 |  |
| Nicotinate and Nicotinamide Metabolism | 6-hydroxynicotinate | GC/MS | [C01020](http://www.genome.jp/dbget-bin/www_bget?cpd+C01020) | [HMDB02658](http://www.hmdb.ca/metabolites/HMDB02658) | 72924 |  |
|  | N1-Methyl-2-pyridone-5-carboxamide | LC/MS Pos | [C05842](http://www.genome.jp/dbget-bin/www_bget?cpd+C05842) | [HMDB04193](http://www.hmdb.ca/metabolites/HMDB04193) | 69698 |  |
|  | nicotinate | GC/MS | [C00253](http://www.genome.jp/dbget-bin/www_bget?cpd+C00253) | [HMDB01488](http://www.hmdb.ca/metabolites/HMDB01488) | 938 |  |
|  | trigonelline (N'-methylnicotinate) | LC/MS Pos | [C01004](http://www.genome.jp/dbget-bin/www_bget?cpd+C01004) | [HMDB00875](http://www.hmdb.ca/metabolites/HMDB00875) | 5570 |  |
| Pantothenate and CoA Metabolism | pantothenate | LC/MS Pos | [C00864](http://www.genome.jp/dbget-bin/www_bget?cpd+C00864) | [HMDB00210](http://www.hmdb.ca/metabolites/HMDB00210) | 6613 |  |
| Riboflavin Metabolism | flavin adenine dinucleotide (FAD) | LC/MS Neg | [C00016](http://www.genome.jp/dbget-bin/www_bget?cpd+C00016) | [HMDB01248](http://www.hmdb.ca/metabolites/HMDB01248) | 643975 |  |
|  | riboflavin (Vitamin B2) | LC/MS Pos | [C00255](http://www.genome.jp/dbget-bin/www_bget?cpd+C00255) | [HMDB00244](http://www.hmdb.ca/metabolites/HMDB00244) | 493570 |  |
| Tetrahydrobiopterin Metabolism | dihydrobiopterin | LC/MS Pos | [C00268](http://www.genome.jp/dbget-bin/www_bget?cpd+C00268) | [HMDB00038](http://www.hmdb.ca/metabolites/HMDB00038) | 1879 |  |
| Thiamine Metabolism | 5-(2-Hydroxyethyl)-4-methylthiazole | GC/MS | [C04294](http://www.genome.jp/dbget-bin/www_bget?cpd+C04294) |  | 1136 |  |
|  | thiamin (Vitamin B1) | LC/MS Pos | [C00378](http://www.genome.jp/dbget-bin/www_bget?cpd+C00378) | [HMDB00325](http://www.hmdb.ca/metabolites/HMDB00325) | 1130 |  |
| Tocopherol Metabolism | alpha-tocopherol | GC/MS | [C02477](http://www.genome.jp/dbget-bin/www_bget?cpd+C02477) | [HMDB01893](http://www.hmdb.ca/metabolites/HMDB01893) | 14985 |  |
|  | delta-tocopherol | GC/MS | [C14151](http://www.genome.jp/dbget-bin/www_bget?cpd+C14151) | [HMDB02902](http://www.hmdb.ca/metabolites/HMDB02902) | 92094 |  |
|  | gamma-tocopherol | GC/MS | [C02483](http://www.genome.jp/dbget-bin/www_bget?cpd+C02483) | [HMDB01492](http://www.hmdb.ca/metabolites/HMDB01492) | 14986 |  |
| Vitamin B6 Metabolism | pyridoxate | LC/MS Neg | [C00847](http://www.genome.jp/dbget-bin/www_bget?cpd+C00847) | [HMDB00017](http://www.hmdb.ca/metabolites/HMDB00017) | 6723 |  |
| Oxidative Phosphorylation | phosphate | GC/MS | [C00009](http://www.genome.jp/dbget-bin/www_bget?cpd+C00009) | [HMDB01429](http://www.hmdb.ca/metabolites/HMDB01429) | 1061 |  |
| TCA Cycle | alpha-ketoglutarate | GC/MS | [C00026](http://www.genome.jp/dbget-bin/www_bget?cpd+C00026) | [HMDB00208](http://www.hmdb.ca/metabolites/HMDB00208) | 51 |  |
|  | citrate | GC/MS | [C00158](http://www.genome.jp/dbget-bin/www_bget?cpd+C00158) | [HMDB00094](http://www.hmdb.ca/metabolites/HMDB00094) | 311 |  |
|  | fumarate | GC/MS | [C00122](http://www.genome.jp/dbget-bin/www_bget?cpd+C00122) | [HMDB00134](http://www.hmdb.ca/metabolites/HMDB00134) | 444972 |  |
|  | malate | GC/MS | [C00149](http://www.genome.jp/dbget-bin/www_bget?cpd+C00149) | [HMDB00156](http://www.hmdb.ca/metabolites/HMDB00156) | 525 |  |
|  | succinate | GC/MS | [C00042](http://www.genome.jp/dbget-bin/www_bget?cpd+C00042) | [HMDB00254](http://www.hmdb.ca/metabolites/HMDB00254) | 1110 |  |
|  | tricarballylate | LC/MS Neg | [C19806](http://www.genome.jp/dbget-bin/www_bget?cpd+C19806) | [HMDB31193](http://www.hmdb.ca/metabolites/HMDB31193) | 14925 |  |
| Carnitine Metabolism | 3-dehydrocarnitine* | LC/MS Pos | [C02636](http://www.genome.jp/dbget-bin/www_bget?cpd+C02636) | [HMDB12154](http://www.hmdb.ca/metabolites/HMDB12154) | 6991982 |  |
|  | carnitine | LC/MS Pos | [C00318](http://www.genome.jp/dbget-bin/www_bget?cpd+C00318) | [HMDB00062](http://www.hmdb.ca/metabolites/HMDB00062) | 10917 |  |
|  | deoxycarnitine | LC/MS Pos | [C01181](http://www.genome.jp/dbget-bin/www_bget?cpd+C01181) | [HMDB01161](http://www.hmdb.ca/metabolites/HMDB01161) | 134 |  |
| Endocannabinoid | oleic ethanolamide | LC/MS Neg |  | [HMDB02088](http://www.hmdb.ca/metabolites/HMDB02088) | 5283454 |  |
|  | palmitoyl ethanolamide | LC/MS Neg | [C16512](http://www.genome.jp/dbget-bin/www_bget?cpd+C16512) | [HMDB02100](http://www.hmdb.ca/metabolites/HMDB02100) | 4671 |  |
| Fatty Acid Metabolism (also BCAA Metabolism) | butyrylglycine | LC/MS Pos |  | [HMDB00808](http://www.hmdb.ca/metabolites/HMDB00808) | 88412 |  |
| Fatty Acid Synthesis | malonate (propanedioate) | LC/MS Neg | [C00383](http://www.genome.jp/dbget-bin/www_bget?cpd+C00383) | [HMDB00691](http://www.hmdb.ca/metabolites/HMDB00691) | 867 |  |
| Fatty Acid, Amino | 2-aminoheptanoate | LC/MS Pos |  |  | 227939 |  |
|  | 2-aminooctanoate | LC/MS Pos |  | [HMDB00991](http://www.hmdb.ca/metabolites/HMDB00991) | 69522 |  |
| Fatty Acid, Branched | 13-methylmyristic acid | GC/MS |  |  | 151014 |  |
|  | 15-methylpalmitate | GC/MS |  |  | 164860 |  |
|  | 17-methylstearate | LC/MS Neg |  |  | 3083779 |  |
|  | isocaproate | LC/MS Neg |  | [HMDB00689](http://www.hmdb.ca/metabolites/HMDB00689) | 12587 |  |
|  | isopalmitic acid | GC/MS |  |  | 36247 |  |
| Fatty Acid, Dicarboxylate | 2-hydroxyadipate | GC/MS | [C02360](http://www.genome.jp/dbget-bin/www_bget?cpd+C02360) | [HMDB00321](http://www.hmdb.ca/metabolites/HMDB00321) | 193530 |  |
|  | 2-hydroxyglutarate | GC/MS | [C02630](http://www.genome.jp/dbget-bin/www_bget?cpd+C02630) | [HMDB00606](http://www.hmdb.ca/metabolites/HMDB00606) | 43 |  |
|  | 2-methylglutarate | GC/MS | [C05282](http://www.genome.jp/dbget-bin/www_bget?cpd+C05282) | [HMDB00752](http://www.hmdb.ca/metabolites/HMDB00752) | 12046 |  |
|  | 3-carboxy-4-methyl-5-propyl-2-furanpropanoate (CMPF) | LC/MS Neg |  | [HMDB61112](http://www.hmdb.ca/metabolites/HMDB61112) | 123979 |  |
|  | adipate | GC/MS | [C06104](http://www.genome.jp/dbget-bin/www_bget?cpd+C06104) | [HMDB00448](http://www.hmdb.ca/metabolites/HMDB00448) | 196 |  |
|  | azelate (nonanedioate) | LC/MS Neg | [C08261](http://www.genome.jp/dbget-bin/www_bget?cpd+C08261) | [HMDB00784](http://www.hmdb.ca/metabolites/HMDB00784) | 2266 |  |
|  | dodecanedioate | LC/MS Neg | [C02678](http://www.genome.jp/dbget-bin/www_bget?cpd+C02678) | [HMDB00623](http://www.hmdb.ca/metabolites/HMDB00623) | 12736 |  |
|  | hexadecanedioate | LC/MS Neg | [C19615](http://www.genome.jp/dbget-bin/www_bget?cpd+C19615) | [HMDB00672](http://www.hmdb.ca/metabolites/HMDB00672) | 10459 |  |
|  | sebacate (decanedioate) | LC/MS Neg | [C08277](http://www.genome.jp/dbget-bin/www_bget?cpd+C08277) | [HMDB00792](http://www.hmdb.ca/metabolites/HMDB00792) | 5192 |  |
|  | suberate (octanedioate) | LC/MS Pos | [C08278](http://www.genome.jp/dbget-bin/www_bget?cpd+C08278) | [HMDB00893](http://www.hmdb.ca/metabolites/HMDB00893) | 10457 |  |
|  | undecanedioate | LC/MS Neg |  | [HMDB00888](http://www.hmdb.ca/metabolites/HMDB00888) | 15816 |  |
| Fatty Acid, Monohydroxy | 10-hydroxyoctadecanoic acid | LC/MS Neg |  |  | 9561835 |  |
|  | 13-HODE + 9-HODE | LC/MS Neg |  |  | 43013 |  |
|  | 2-hydroxydecanoate | LC/MS Neg |  |  | 21488 |  |
|  | 2-hydroxyoctanoate | LC/MS Neg |  | [HMDB02264](http://www.hmdb.ca/metabolites/HMDB02264) | 94180 |  |
|  | 2-hydroxypalmitate | LC/MS Neg |  | [HMDB31057](http://www.hmdb.ca/metabolites/HMDB31057) | 92836 |  |
|  | 2-hydroxystearate | LC/MS Neg | [C03045](http://www.genome.jp/dbget-bin/www_bget?cpd+C03045) |  | 69417 |  |
|  | 3-hydroxymyristate | LC/MS Neg |  |  | 16064 |  |
|  | 3-hydroxypropanoate | GC/MS | [C01013](http://www.genome.jp/dbget-bin/www_bget?cpd+C01013) | [HMDB00700](http://www.hmdb.ca/metabolites/HMDB00700) | 68152 |  |
|  | 4-hydroxybutyrate (GHB) | GC/MS | [C00989](http://www.genome.jp/dbget-bin/www_bget?cpd+C00989) | [HMDB00710](http://www.hmdb.ca/metabolites/HMDB00710) | 10413 |  |
|  | 5-hydroxyhexanoate | GC/MS |  | [HMDB00525](http://www.hmdb.ca/metabolites/HMDB00525) | 170748 |  |
|  | 8-hydroxyoctanoate | LC/MS Neg |  | [HMDB00711](http://www.hmdb.ca/metabolites/HMDB00711) | 69820 |  |
| Fatty Alcohol, Long Chain | 1-hexadecanol | GC/MS | [C00823](http://www.genome.jp/dbget-bin/www_bget?cpd+C00823) | [HMDB03424](http://www.hmdb.ca/metabolites/HMDB03424) | 2682 |  |
|  | 1-octadecanol | GC/MS | [D01924](http://www.genome.jp/dbget-bin/www_bget?cpd+D01924) | [HMDB02350](http://www.hmdb.ca/metabolites/HMDB02350) | 8221 |  |
| Glycerolipid Metabolism | glycerol | GC/MS | [C00116](http://www.genome.jp/dbget-bin/www_bget?cpd+C00116) | [HMDB00131](http://www.hmdb.ca/metabolites/HMDB00131) | 753 |  |
|  | glycerol 3-phosphate (G3P) | GC/MS | [C00093](http://www.genome.jp/dbget-bin/www_bget?cpd+C00093) | [HMDB00126](http://www.hmdb.ca/metabolites/HMDB00126) | 754 |  |
| Inositol Metabolism | inositol 1-phosphate (I1P) | GC/MS | [C04006](http://www.genome.jp/dbget-bin/www_bget?cpd+C04006) | [HMDB00213](http://www.hmdb.ca/metabolites/HMDB00213) | 440194 |  |
|  | myo-inositol | GC/MS | [C00137](http://www.genome.jp/dbget-bin/www_bget?cpd+C00137) | [HMDB00211](http://www.hmdb.ca/metabolites/HMDB00211) | 892 |  |
| Ketone Bodies | 3-hydroxybutyrate (BHBA) | GC/MS | [C01089](http://www.genome.jp/dbget-bin/www_bget?cpd+C01089) | [HMDB00357](http://www.hmdb.ca/metabolites/HMDB00357) | 441 |  |
| Long Chain Fatty Acid | 10-heptadecenoate (17:1n7) | LC/MS Neg |  |  | 5312435 |  |
|  | 10-nonadecenoate (19:1n9) | LC/MS Neg |  | [HMDB13622](http://www.hmdb.ca/metabolites/HMDB13622) | 5312513 |  |
|  | arachidate (20:0) | LC/MS Neg | [C06425](http://www.genome.jp/dbget-bin/www_bget?cpd+C06425) | [HMDB02212](http://www.hmdb.ca/metabolites/HMDB02212) | 10467 |  |
|  | behenate (22:0) | GC/MS | [C08281](http://www.genome.jp/dbget-bin/www_bget?cpd+C08281) | [HMDB00944](http://www.hmdb.ca/metabolites/HMDB00944) | 8215 |  |
|  | cis-vaccenate (18:1n7) | GC/MS | [C08367](http://www.genome.jp/dbget-bin/www_bget?cpd+C08367) | [HMDB03231](http://www.hmdb.ca/metabolites/HMDB03231) | 5282761 |  |
|  | eicosenoate (20:1n9 or 11) | LC/MS Neg |  |  | 5282768 |  |
|  | erucate (22:1n9) | LC/MS Neg | [C08316](http://www.genome.jp/dbget-bin/www_bget?cpd+C08316) | [HMDB02068](http://www.hmdb.ca/metabolites/HMDB02068) | 5281116 |  |
|  | lignocerate (24:0) | GC/MS | [C08320](http://www.genome.jp/dbget-bin/www_bget?cpd+C08320) | [HMDB02003](http://www.hmdb.ca/metabolites/HMDB02003) | 11197 |  |
|  | margarate (17:0) | LC/MS Neg |  | [HMDB02259](http://www.hmdb.ca/metabolites/HMDB02259) | 10465 |  |
|  | myristate (14:0) | LC/MS Neg | [C06424](http://www.genome.jp/dbget-bin/www_bget?cpd+C06424) | [HMDB00806](http://www.hmdb.ca/metabolites/HMDB00806) | 11005 |  |
|  | myristoleate (14:1n5) | LC/MS Neg | [C08322](http://www.genome.jp/dbget-bin/www_bget?cpd+C08322) | [HMDB02000](http://www.hmdb.ca/metabolites/HMDB02000) | 5281119 |  |
|  | nervonate (24:1n9) | GC/MS | [C08323](http://www.genome.jp/dbget-bin/www_bget?cpd+C08323) | [HMDB02368](http://www.hmdb.ca/metabolites/HMDB02368) | 5281120 |  |
|  | nonadecanoate (19:0) | LC/MS Neg | [C16535](http://www.genome.jp/dbget-bin/www_bget?cpd+C16535) | [HMDB00772](http://www.hmdb.ca/metabolites/HMDB00772) | 12591 |  |
|  | oleate (18:1n9) | GC/MS | [C00712](http://www.genome.jp/dbget-bin/www_bget?cpd+C00712) | [HMDB00207](http://www.hmdb.ca/metabolites/HMDB00207) | 445639 |  |
|  | palmitate (16:0) | LC/MS Neg | [C00249](http://www.genome.jp/dbget-bin/www_bget?cpd+C00249) | [HMDB00220](http://www.hmdb.ca/metabolites/HMDB00220) | 985 |  |
|  | palmitoleate (16:1n7) | LC/MS Neg | [C08362](http://www.genome.jp/dbget-bin/www_bget?cpd+C08362) | [HMDB03229](http://www.hmdb.ca/metabolites/HMDB03229) | 445638 |  |
|  | pentadecanoate (15:0) | GC/MS | [C16537](http://www.genome.jp/dbget-bin/www_bget?cpd+C16537) | [HMDB00826](http://www.hmdb.ca/metabolites/HMDB00826) | 13849 |  |
|  | stearate (18:0) | LC/MS Neg | [C01530](http://www.genome.jp/dbget-bin/www_bget?cpd+C01530) | [HMDB00827](http://www.hmdb.ca/metabolites/HMDB00827) | 5281 |  |
| Lysolipid | 1-linoleoylglycerophosphoethanolamine* | LC/MS Neg |  | [HMDB11507](http://www.hmdb.ca/metabolites/HMDB11507) | 52925130 |  |
|  | 1-oleoylglycerophosphocholine (18:1) | LC/MS Pos |  |  | 16081932 |  |
|  | 1-oleoylglycerophosphoethanolamine | LC/MS Neg |  | [HMDB11506](http://www.hmdb.ca/metabolites/HMDB11506) | 9547071 |  |
|  | 1-palmitoylglycerophosphocholine (16:0) | LC/MS Pos |  |  | 86554 |  |
|  | 1-palmitoylglycerophosphoethanolamine | LC/MS Neg |  | [HMDB11503](http://www.hmdb.ca/metabolites/HMDB11503) | 9547069 |  |
|  | 1-palmitoylglycerophosphoglycerol* | LC/MS Neg |  |  | 3300276 |  |
|  | 1-palmitoylglycerophosphoinositol* | LC/MS Neg |  |  |  |  |
|  | 1-palmitoylplasmenylethanolamine* | LC/MS Neg |  |  |  |  |
|  | 1-stearoylglycerophosphocholine (18:0) | LC/MS Pos |  |  | 497299 |  |
|  | 1-stearoylglycerophosphoethanolamine | LC/MS Pos |  | [HMDB11130](http://www.hmdb.ca/metabolites/HMDB11130) | 9547068 |  |
|  | 1-stearoylglycerophosphoglycerol | LC/MS Neg |  |  |  |  |
|  | 1-stearoylglycerophosphoinositol | LC/MS Neg |  |  |  |  |
|  | 1-stearoylglycerophosphoserine* | LC/MS Neg |  |  | 9547101 |  |
|  | 1-stearoylplasmenylethanolamine* | LC/MS Neg |  |  |  |  |
|  | 2-palmitoylglycerophosphocholine* | LC/MS Pos |  |  | 15061532 |  |
|  | 2-palmitoylglycerophosphoethanolamine* | LC/MS Pos |  |  |  |  |
| Medium Chain Fatty Acid | caprate (10:0) | LC/MS Neg | [C01571](http://www.genome.jp/dbget-bin/www_bget?cpd+C01571) | [HMDB00511](http://www.hmdb.ca/metabolites/HMDB00511) | 2969 |  |
|  | caproate (6:0) | LC/MS Neg | [C01585](http://www.genome.jp/dbget-bin/www_bget?cpd+C01585) | [HMDB00535](http://www.hmdb.ca/metabolites/HMDB00535) | 8892 |  |
|  | caprylate (8:0) | LC/MS Neg | [C06423](http://www.genome.jp/dbget-bin/www_bget?cpd+C06423) | [HMDB00482](http://www.hmdb.ca/metabolites/HMDB00482) | 379 |  |
|  | heptanoate (7:0) | LC/MS Neg | [C17714](http://www.genome.jp/dbget-bin/www_bget?cpd+C17714) | [HMDB00666](http://www.hmdb.ca/metabolites/HMDB00666) | 8094 |  |
|  | laurate (12:0) | LC/MS Neg | [C02679](http://www.genome.jp/dbget-bin/www_bget?cpd+C02679) | [HMDB00638](http://www.hmdb.ca/metabolites/HMDB00638) | 3893 |  |
|  | pelargonate (9:0) | LC/MS Neg | [C01601](http://www.genome.jp/dbget-bin/www_bget?cpd+C01601) | [HMDB00847](http://www.hmdb.ca/metabolites/HMDB00847) | 8158 |  |
| Monoacylglycerol | 1-linolenoylglycerol | LC/MS Neg |  | [HMDB11569](http://www.hmdb.ca/metabolites/HMDB11569) | 53480978 |  |
|  | 1-linoleoylglycerol (1-monolinolein) | GC/MS |  |  | 5283469 |  |
|  | 1-oleoylglycerol (1-monoolein) | LC/MS Neg |  | [HMDB11567](http://www.hmdb.ca/metabolites/HMDB11567) | 5283468 |  |
|  | 1-palmitoylglycerol (1-monopalmitin) | GC/MS |  | [HMDB31074](http://www.hmdb.ca/metabolites/HMDB31074) | 14900 |  |
|  | 1-pentadecanoylglycerol (1-monopentadecanoin) | LC/MS Neg |  |  | 190750 |  |
|  | 1-stearoylglycerol (1-monostearin) | GC/MS | [D01947](http://www.genome.jp/dbget-bin/www_bget?cpd+D01947) | [HMDB31075](http://www.hmdb.ca/metabolites/HMDB31075) | 24699 |  |
|  | 2-linoleoylglycerol (2-monolinolein) | GC/MS |  | [HMDB11538](http://www.hmdb.ca/metabolites/HMDB11538) | 5365676 |  |
|  | 2-oleoylglycerol (2-monoolein) | GC/MS |  |  | 5319879 |  |
| Phospholipid Metabolism | ethanolamine | GC/MS | [C00189](http://www.genome.jp/dbget-bin/www_bget?cpd+C00189) | [HMDB00149](http://www.hmdb.ca/metabolites/HMDB00149) | 700 |  |
| Polyunsaturated Fatty Acid (n3 and n6) | arachidonate (20:4n6) | LC/MS Neg | [C00219](http://www.genome.jp/dbget-bin/www_bget?cpd+C00219) | [HMDB01043](http://www.hmdb.ca/metabolites/HMDB01043) | 444899 |  |
|  | dihomo-linoleate (20:2n6) | LC/MS Neg | [C16525](http://www.genome.jp/dbget-bin/www_bget?cpd+C16525) | [HMDB05060](http://www.hmdb.ca/metabolites/HMDB05060) | 6439848 |  |
|  | dihomo-linolenate (20:3n3 or n6) | LC/MS Neg | [C03242](http://www.genome.jp/dbget-bin/www_bget?cpd+C03242) | [HMDB02925](http://www.hmdb.ca/metabolites/HMDB02925) | 5280581 |  |
|  | docosadienoate (22:2n6) | LC/MS Neg | [C16533](http://www.genome.jp/dbget-bin/www_bget?cpd+C16533) |  | 5282807 |  |
|  | docosahexaenoate (DHA; 22:6n3) | LC/MS Neg | [C06429](http://www.genome.jp/dbget-bin/www_bget?cpd+C06429) | [HMDB02183](http://www.hmdb.ca/metabolites/HMDB02183) | 445580 |  |
|  | docosapentaenoate (n3 DPA; 22:5n3) | LC/MS Neg | [C16513](http://www.genome.jp/dbget-bin/www_bget?cpd+C16513) | [HMDB01976](http://www.hmdb.ca/metabolites/HMDB01976) | 6441454 |  |
|  | docosapentaenoate (n6 DPA; 22:5n6) | LC/MS Neg | [C16513](http://www.genome.jp/dbget-bin/www_bget?cpd+C16513) | [HMDB13123](http://www.hmdb.ca/metabolites/HMDB13123) | 6441454 |  |
|  | eicosapentaenoate (EPA; 20:5n3) | LC/MS Neg | [C06428](http://www.genome.jp/dbget-bin/www_bget?cpd+C06428) | [HMDB01999](http://www.hmdb.ca/metabolites/HMDB01999) | 446284 |  |
|  | linoleate (18:2n6) | LC/MS Neg | [C01595](http://www.genome.jp/dbget-bin/www_bget?cpd+C01595) | [HMDB00673](http://www.hmdb.ca/metabolites/HMDB00673) | 5280450 |  |
|  | linolenate [alpha or gamma; (18:3n3 or 6)] | LC/MS Neg | [C06427](http://www.genome.jp/dbget-bin/www_bget?cpd+C06427) |  | 5280934 |  |
|  | mead acid (20:3n9) | LC/MS Neg |  | [HMDB10378](http://www.hmdb.ca/metabolites/HMDB10378) | 5312531 |  |
| Primary Bile Acid Metabolism | beta-muricholate | LC/MS Pos | [C17726](http://www.genome.jp/dbget-bin/www_bget?cpd+C17726) | [HMDB00415](http://www.hmdb.ca/metabolites/HMDB00415) | 5283853 |  |
|  | cholate | LC/MS Pos | [C00695](http://www.genome.jp/dbget-bin/www_bget?cpd+C00695) | [HMDB00619](http://www.hmdb.ca/metabolites/HMDB00619) | 221493 |  |
|  | glycochenodeoxycholate | LC/MS Neg | [C05466](http://www.genome.jp/dbget-bin/www_bget?cpd+C05466) | [HMDB00637](http://www.hmdb.ca/metabolites/HMDB00637) | 12544 |  |
|  | glycocholate | LC/MS Neg | [C01921](http://www.genome.jp/dbget-bin/www_bget?cpd+C01921) | [HMDB00138](http://www.hmdb.ca/metabolites/HMDB00138) | 10140 |  |
|  | taurochenodeoxycholate | LC/MS Neg | [C05465](http://www.genome.jp/dbget-bin/www_bget?cpd+C05465) | [HMDB00951](http://www.hmdb.ca/metabolites/HMDB00951) | 387316 |  |
|  | taurocholate | LC/MS Neg | [C05122](http://www.genome.jp/dbget-bin/www_bget?cpd+C05122) | [HMDB00036](http://www.hmdb.ca/metabolites/HMDB00036) | 6675 |  |
| Secondary Bile Acid Metabolism | 12-dehydrocholate | LC/MS Neg |  | [HMDB00400](http://www.hmdb.ca/metabolites/HMDB00400) | 94235 |  |
|  | 3-dehydrocholate | LC/MS Pos |  | [HMDB00502](http://www.hmdb.ca/metabolites/HMDB00502) | 159655 |  |
|  | 7,12-diketolithocholate | LC/MS Neg |  |  | 3080560 |  |
|  | 7-ketodeoxycholate | LC/MS Neg |  | [HMDB00391](http://www.hmdb.ca/metabolites/HMDB00391) | 188292 |  |
|  | deoxycholate | GC/MS | [C04483](http://www.genome.jp/dbget-bin/www_bget?cpd+C04483) | [HMDB00626](http://www.hmdb.ca/metabolites/HMDB00626) | 222528 |  |
|  | glycocholenate sulfate* | LC/MS Neg |  |  |  |  |
|  | glycodeoxycholate | LC/MS Neg | [C05464](http://www.genome.jp/dbget-bin/www_bget?cpd+C05464) | [HMDB00631](http://www.hmdb.ca/metabolites/HMDB00631) | 3035026 |  |
|  | glycolithocholate sulfate* | LC/MS Neg | [C11301](http://www.genome.jp/dbget-bin/www_bget?cpd+C11301) | [HMDB02639](http://www.hmdb.ca/metabolites/HMDB02639) | 72222 |  |
|  | lithocholate | GC/MS | [C03990](http://www.genome.jp/dbget-bin/www_bget?cpd+C03990) | [HMDB00761](http://www.hmdb.ca/metabolites/HMDB00761) | 9903 |  |
|  | lithocholate [6-oxo or 7-keto] | LC/MS Pos |  |  | 53477693 |  |
|  | taurodeoxycholate | LC/MS Neg | [C05463](http://www.genome.jp/dbget-bin/www_bget?cpd+C05463) | [HMDB00896](http://www.hmdb.ca/metabolites/HMDB00896) | 2733768 |  |
|  | tauroursodeoxycholate | LC/MS Neg |  | [HMDB00874](http://www.hmdb.ca/metabolites/HMDB00874) | 9848818 |  |
| Short Chain Fatty Acid | valerate | LC/MS Neg | [C00803](http://www.genome.jp/dbget-bin/www_bget?cpd+C00803) | [HMDB00892](http://www.hmdb.ca/metabolites/HMDB00892) | 7991 |  |
| Sphingolipid Metabolism | N-acetylsphingosine | LC/MS Neg | [C00195](http://www.genome.jp/dbget-bin/www_bget?cpd+C00195) | [HMDB04950,HMDB04952,HMDB04949,HMDB04955,HMDB04953,HMDB04947,HMDB04954,HMDB04948,HMDB04951,HMDB04956,HMDB04957](http://www.hmdb.ca/metabolites/HMDB04950,HMDB04952,HMDB04949,HMDB04955,HMDB04953,HMDB04947,HMDB04954,HMDB04948,HMDB04951,HMDB04956,HMDB04957) | 5497136 |  |
|  | palmitoyl sphingomyelin | GC/MS |  |  | 9939941 |  |
|  | sphinganine | LC/MS Pos | [C00836](http://www.genome.jp/dbget-bin/www_bget?cpd+C00836) | [HMDB00269](http://www.hmdb.ca/metabolites/HMDB00269) | 3126 |  |
|  | sphingosine | LC/MS Pos | [C00319](http://www.genome.jp/dbget-bin/www_bget?cpd+C00319) | [HMDB00252](http://www.hmdb.ca/metabolites/HMDB00252) | 5353955 |  |
| Steroid | 4-androsten-3beta,17beta-diol disulfate (1)* | LC/MS Neg | [C04295](http://www.genome.jp/dbget-bin/www_bget?cpd+C04295) | [HMDB03818](http://www.hmdb.ca/metabolites/HMDB03818) | 10634 |  |
|  | 4-androsten-3beta,17beta-diol disulfate (2)* | LC/MS Neg | [C04295](http://www.genome.jp/dbget-bin/www_bget?cpd+C04295) | [HMDB03818](http://www.hmdb.ca/metabolites/HMDB03818) | 10634 |  |
|  | 5alpha-androstan-3alpha,17beta-diol disulfate | LC/MS Neg |  |  |  |  |
|  | 5alpha-pregnan-3beta,20alpha-diol disulfate | LC/MS Neg |  |  |  |  |
|  | androsterone sulfate | LC/MS Neg |  | [HMDB02759](http://www.hmdb.ca/metabolites/HMDB02759) | 159663 |  |
|  | dehydroisoandrosterone sulfate (DHEA-S) | LC/MS Neg | [C04555](http://www.genome.jp/dbget-bin/www_bget?cpd+C04555) | [HMDB01032](http://www.hmdb.ca/metabolites/HMDB01032) | 12594 |  |
|  | epiandrosterone sulfate | LC/MS Neg | [C07635](http://www.genome.jp/dbget-bin/www_bget?cpd+C07635) | [HMDB00365](http://www.hmdb.ca/metabolites/HMDB00365) |  |  |
|  | pregn steroid monosulfate* | LC/MS Neg |  |  |  |  |
|  | pregnen-diol disulfate* | LC/MS Neg |  |  |  |  |
| Sterol | 7-alpha-hydroxycholesterol | GC/MS | [C03594](http://www.genome.jp/dbget-bin/www_bget?cpd+C03594) | [HMDB01496](http://www.hmdb.ca/metabolites/HMDB01496) | 107722 |  |
|  | 7-beta-hydroxycholesterol | GC/MS |  | [HMDB06119](http://www.hmdb.ca/metabolites/HMDB06119) | 473141 |  |
|  | beta-sitosterol | GC/MS | [C01753](http://www.genome.jp/dbget-bin/www_bget?cpd+C01753) | [HMDB00852](http://www.hmdb.ca/metabolites/HMDB00852) | 222284 |  |
|  | campesterol | GC/MS | [C01789](http://www.genome.jp/dbget-bin/www_bget?cpd+C01789) | [HMDB02869](http://www.hmdb.ca/metabolites/HMDB02869) | 173183 |  |
|  | cholesterol | GC/MS | [C00187](http://www.genome.jp/dbget-bin/www_bget?cpd+C00187) | [HMDB00067](http://www.hmdb.ca/metabolites/HMDB00067) | 11025495 |  |
|  | coprostanol | GC/MS |  |  |  |  |
|  | squalene | GC/MS | [C00751](http://www.genome.jp/dbget-bin/www_bget?cpd+C00751) | [HMDB00256](http://www.hmdb.ca/metabolites/HMDB00256) | 638072 |  |
| Purine and Pyrimidine Metabolism | methylphosphate | GC/MS |  |  | 13130 |  |
| Purine Metabolism, (Hypo)Xanthine/Inosine containing | 2'-deoxyinosine | LC/MS Neg | [C05512](http://www.genome.jp/dbget-bin/www_bget?cpd+C05512) | [HMDB00071](http://www.hmdb.ca/metabolites/HMDB00071) | 65058 |  |
|  | hypoxanthine | LC/MS Neg | [C00262](http://www.genome.jp/dbget-bin/www_bget?cpd+C00262) | [HMDB00157](http://www.hmdb.ca/metabolites/HMDB00157) | 790 |  |
|  | inosine | GC/MS | [C00294](http://www.genome.jp/dbget-bin/www_bget?cpd+C00294) | [HMDB00195](http://www.hmdb.ca/metabolites/HMDB00195) | 6021 |  |
|  | urate | GC/MS | [C00366](http://www.genome.jp/dbget-bin/www_bget?cpd+C00366) | [HMDB00289](http://www.hmdb.ca/metabolites/HMDB00289) | 1175 |  |
|  | xanthine | GC/MS | [C00385](http://www.genome.jp/dbget-bin/www_bget?cpd+C00385) | [HMDB00292](http://www.hmdb.ca/metabolites/HMDB00292) | 1188 |  |
| Purine Metabolism, Adenine containing | adenine | LC/MS Pos | [C00147](http://www.genome.jp/dbget-bin/www_bget?cpd+C00147) | [HMDB00034](http://www.hmdb.ca/metabolites/HMDB00034) | 190 |  |
|  | adenosine-2',3'-cyclic monophosphate | LC/MS Neg | [C02353](http://www.genome.jp/dbget-bin/www_bget?cpd+C02353) | [HMDB11616](http://www.hmdb.ca/metabolites/HMDB11616) | 2024 |  |
| Purine Metabolism, Guanine containing | 2'-deoxyguanosine | LC/MS Neg | [C00330](http://www.genome.jp/dbget-bin/www_bget?cpd+C00330) | [HMDB00085](http://www.hmdb.ca/metabolites/HMDB00085) | 187790 |  |
|  | 8-hydroxyguanine | GC/MS | [C20155](http://www.genome.jp/dbget-bin/www_bget?cpd+C20155) | [HMDB02032](http://www.hmdb.ca/metabolites/HMDB02032) | 65154 |  |
|  | guanine | GC/MS | [C00242](http://www.genome.jp/dbget-bin/www_bget?cpd+C00242) | [HMDB00132](http://www.hmdb.ca/metabolites/HMDB00132) | 764 |  |
|  | guanosine | LC/MS Neg | [C00387](http://www.genome.jp/dbget-bin/www_bget?cpd+C00387) | [HMDB00133](http://www.hmdb.ca/metabolites/HMDB00133) | 6802 |  |
| Pyrimidine Metabolism, Cytidine containing | cytidine | LC/MS Pos | [C00475](http://www.genome.jp/dbget-bin/www_bget?cpd+C00475) | [HMDB00089](http://www.hmdb.ca/metabolites/HMDB00089) | 6175 |  |
| Pyrimidine Metabolism, Orotate containing | orotate | GC/MS | [C00295](http://www.genome.jp/dbget-bin/www_bget?cpd+C00295) | [HMDB00226](http://www.hmdb.ca/metabolites/HMDB00226) | 967 |  |
| Pyrimidine Metabolism, Thymine containing | 3-aminoisobutyrate | GC/MS | [C05145](http://www.genome.jp/dbget-bin/www_bget?cpd+C05145) | [HMDB03911](http://www.hmdb.ca/metabolites/HMDB03911) | 64956 |  |
|  | thymidine | LC/MS Neg | [C00214](http://www.genome.jp/dbget-bin/www_bget?cpd+C00214) | [HMDB00273](http://www.hmdb.ca/metabolites/HMDB00273) | 5789 |  |
|  | thymidine 5'-monophosphate | LC/MS Neg | [C00364](http://www.genome.jp/dbget-bin/www_bget?cpd+C00364) | [HMDB01227](http://www.hmdb.ca/metabolites/HMDB01227) | 9700 |  |
|  | thymine | GC/MS | [C00178](http://www.genome.jp/dbget-bin/www_bget?cpd+C00178) | [HMDB00262](http://www.hmdb.ca/metabolites/HMDB00262) | 1135 |  |
| Pyrimidine Metabolism, Uracil containing | 2'-deoxyuridine | LC/MS Neg | [C00526](http://www.genome.jp/dbget-bin/www_bget?cpd+C00526) | [HMDB00012](http://www.hmdb.ca/metabolites/HMDB00012) | 13712 |  |
|  | 3-ureidopropionate | LC/MS Pos | [C02642](http://www.genome.jp/dbget-bin/www_bget?cpd+C02642) | [HMDB00026](http://www.hmdb.ca/metabolites/HMDB00026) | 111 |  |
|  | 5-methyluridine (ribothymidine) | LC/MS Neg |  | [HMDB00884](http://www.hmdb.ca/metabolites/HMDB00884) | 445408 |  |
|  | beta-alanine | GC/MS | [C00099](http://www.genome.jp/dbget-bin/www_bget?cpd+C00099) | [HMDB00056](http://www.hmdb.ca/metabolites/HMDB00056) | 239 |  |
|  | pseudouridine | LC/MS Neg | [C02067](http://www.genome.jp/dbget-bin/www_bget?cpd+C02067) | [HMDB00767](http://www.hmdb.ca/metabolites/HMDB00767) | 15047 |  |
|  | uracil | GC/MS | [C00106](http://www.genome.jp/dbget-bin/www_bget?cpd+C00106) | [HMDB00300](http://www.hmdb.ca/metabolites/HMDB00300) | 1174 |  |
|  | uridine | LC/MS Neg | [C00299](http://www.genome.jp/dbget-bin/www_bget?cpd+C00299) | [HMDB00296](http://www.hmdb.ca/metabolites/HMDB00296) | 6029 |  |
| Dipeptide | alanylalanine | LC/MS Pos |  | [HMDB28680](http://www.hmdb.ca/metabolites/HMDB28680) | 5484352 |  |
|  | alanylglutamate | LC/MS Neg |  |  | 656476 |  |
|  | alanylisoleucine | LC/MS Pos |  |  | 417358;5246008 |  |
|  | alanylleucine | LC/MS Pos |  |  | 259583 |  |
|  | alanylphenylalanine | LC/MS Pos |  |  | 2080 |  |
|  | alanylproline | LC/MS Pos |  |  | 418040 |  |
|  | alanyltryptophan | LC/MS Pos |  |  | 1550278;85362 |  |
|  | alanylvaline | LC/MS Pos |  |  | 137276 |  |
|  | alpha-glutamyltryptophan | LC/MS Pos |  |  | 333386 |  |
|  | alpha-glutamyltyrosine | LC/MS Pos |  |  | 351830 |  |
|  | arginylleucine | LC/MS Pos |  |  | 333445 |  |
|  | arginylphenylalanine | LC/MS Pos |  |  | 4441256 |  |
|  | arginylvaline | LC/MS Pos |  |  | 335257 |  |
|  | asparagylisoleucine | LC/MS Pos |  |  |  |  |
|  | asparagylleucine | LC/MS Pos |  |  |  |  |
|  | asparagylvaline | LC/MS Pos |  |  | 7019993 |  |
|  | aspartylleucine | LC/MS Pos |  |  | 332962 |  |
|  | aspartylphenylalanine | LC/MS Pos |  | [HMDB00706](http://www.hmdb.ca/metabolites/HMDB00706) | 93078 |  |
|  | cyclo(leu-phe) | LC/MS Pos |  |  | 7076347 |  |
|  | glutamine-isoleucine | LC/MS Pos |  |  |  |  |
|  | glutamine-leucine | LC/MS Pos |  |  |  |  |
|  | glycylglycine | GC/MS | [C02037](http://www.genome.jp/dbget-bin/www_bget?cpd+C02037) | [HMDB11733](http://www.hmdb.ca/metabolites/HMDB11733) | 11163 |  |
|  | glycylisoleucine | LC/MS Pos |  |  | 88079 |  |
|  | glycylleucine | LC/MS Pos | [C02155](http://www.genome.jp/dbget-bin/www_bget?cpd+C02155) | [HMDB00759](http://www.hmdb.ca/metabolites/HMDB00759) | 92843 |  |
|  | glycylmethionine | LC/MS Pos |  |  | 96757 |  |
|  | glycylphenylalanine | LC/MS Pos |  | [HMDB28848](http://www.hmdb.ca/metabolites/HMDB28848) | 92953 |  |
|  | glycylproline | LC/MS Pos |  | [HMDB00721](http://www.hmdb.ca/metabolites/HMDB00721) | 3013625 |  |
|  | glycylthreonine | GC/MS |  |  | 351980 |  |
|  | glycyltryptophan | LC/MS Pos |  |  | 1551340;92181 |  |
|  | glycyltyrosine | LC/MS Pos |  | [HMDB28853](http://www.hmdb.ca/metabolites/HMDB28853) | 92829 |  |
|  | glycylvaline | LC/MS Pos |  | [HMDB28854](http://www.hmdb.ca/metabolites/HMDB28854) | 97417 |  |
|  | histidylisoleucine | LC/MS Pos |  |  | 4060215 |  |
|  | histidylleucine | LC/MS Pos |  |  | 189008;6992010 |  |
|  | histidylphenylalanine | LC/MS Pos |  |  | 4466133 |  |
|  | histidylproline | LC/MS Pos |  |  |  |  |
|  | histidylvaline | LC/MS Pos |  |  | 7021871 |  |
|  | isoleucylalanine | LC/MS Pos |  |  | 5246009;5246010 |  |
|  | isoleucylarginine | LC/MS Pos |  |  | 9900561 |  |
|  | isoleucylglutamate | LC/MS Pos |  |  |  |  |
|  | isoleucylglycine | LC/MS Pos |  |  | 342532 |  |
|  | isoleucylisoleucine | LC/MS Pos |  |  | 7010568 |  |
|  | isoleucylleucine | LC/MS Pos |  |  | 11644431 |  |
|  | isoleucylmethionine | LC/MS Pos |  |  | 7020106 |  |
|  | isoleucylphenylalanine | LC/MS Pos |  |  | 435728 |  |
|  | isoleucylserine | LC/MS Neg |  |  | 7021816 |  |
|  | isoleucylthreonine | LC/MS Pos |  |  | 16122515 |  |
|  | isoleucyltryptophan | LC/MS Pos |  |  | 9797038 |  |
|  | isoleucyltyrosine | LC/MS Neg |  |  | 342468 |  |
|  | isoleucylvaline | LC/MS Pos |  |  | 5246011;435949 |  |
|  | leucylalanine | LC/MS Pos |  |  | 259321 |  |
|  | leucylglutamate | LC/MS Pos |  |  | 5259589;5259590 |  |
|  | leucylglycine | LC/MS Pos |  |  | 79070 |  |
|  | leucylisoleucine | LC/MS Pos |  |  | 7010534 |  |
|  | leucylmethionine | LC/MS Pos |  |  | 118276 |  |
|  | leucylphenylalanine | LC/MS Pos |  |  | 259325 |  |
|  | leucylserine | LC/MS Neg |  |  | 3621685 |  |
|  | leucylthreonine | LC/MS Pos |  |  | 10353878 |  |
|  | leucyltryptophan | LC/MS Pos |  |  | 329275 |  |
|  | leucyltyrosine | LC/MS Neg |  |  | 273262 |  |
|  | lysylisoleucine | LC/MS Pos |  |  |  |  |
|  | lysylleucine | LC/MS Pos |  |  | 4682588 |  |
|  | lysylvaline | LC/MS Neg |  |  | 140709 |  |
|  | methionylvaline | LC/MS Pos |  |  | 7010520 |  |
|  | phenylacetylphenylalanine | LC/MS Neg |  |  | 47583 |  |
|  | phenylalanylalanine | LC/MS Neg |  |  | 6993123;5488196 |  |
|  | phenylalanylaspartate | LC/MS Pos |  |  | 335051 |  |
|  | phenylalanylglutamate | LC/MS Pos |  |  | 4422358 |  |
|  | phenylalanylisoleucine | LC/MS Pos |  |  | 7010566 |  |
|  | phenylalanylleucine | LC/MS Pos |  |  | 4078229 |  |
|  | phenylalanylphenylalanine | LC/MS Pos |  |  | 6993090;6993089 |  |
|  | phenylalanylserine | LC/MS Neg |  |  | 9859812 |  |
|  | phenylalanylvaline | LC/MS Pos |  |  | 4096934 |  |
|  | pyroglutamylvaline | LC/MS Neg |  |  | 152416 |  |
|  | serylisoleucine* | LC/MS Pos |  |  | 71429009 |  |
|  | serylleucine | LC/MS Pos |  |  | 7015695 |  |
|  | serylphenyalanine | LC/MS Pos |  |  |  |  |
|  | serylvaline | LC/MS Pos |  |  | 7020159 |  |
|  | threonylisoleucine | LC/MS Pos |  |  |  |  |
|  | threonylleucine | LC/MS Pos |  |  | 4420322 |  |
|  | threonylphenylalanine | LC/MS Pos |  |  | 4099799;4099798 |  |
|  | threonylvaline | LC/MS Pos |  |  | 416721;4278432 |  |
|  | tryptophylglycine | LC/MS Pos |  |  | 263471 |  |
|  | tryptophylisoleucine | LC/MS Pos |  |  | 47349 |  |
|  | tryptophylleucine | LC/MS Pos |  |  | 323786 |  |
|  | tryptophylvaline | LC/MS Pos |  |  | 7009658;7009657 |  |
|  | tyrosylisoleucine | LC/MS Pos |  |  | 7019110 |  |
|  | tyrosylleucine | LC/MS Pos |  |  | 87071;7009561 |  |
|  | tyrosylvaline | LC/MS Pos |  |  | 7009560;7009559 |  |
|  | valylalanine | LC/MS Pos |  |  | 334517 |  |
|  | valylaspartate | LC/MS Pos |  |  | 9964657 |  |
|  | valylglutamate | LC/MS Pos |  |  | 7009623 |  |
|  | valylisoleucine | LC/MS Pos |  |  | 5246012;5246013 |  |
|  | valylleucine | LC/MS Pos |  |  | 352039 |  |
|  | valyllysine | LC/MS Neg |  |  | 5253210 |  |
|  | valylmethionine | LC/MS Pos |  |  | 292427 |  |
|  | valylphenylalanine | LC/MS Neg |  |  | 6993119;6993120 |  |
|  | valylthreonine | LC/MS Pos |  |  | 4046492 |  |
|  | valyltyrosine | LC/MS Neg |  |  | 4065033 |  |
|  | valylvaline | LC/MS Neg |  |  | 409682;4280382 |  |
| Gamma-glutamyl Amino Acid | gamma-glutamylglutamate | LC/MS Pos | [C05282](http://www.genome.jp/dbget-bin/www_bget?cpd+C05282) | [HMDB11737](http://www.hmdb.ca/metabolites/HMDB11737) | 92865 |  |
|  | gamma-glutamylisoleucine* | LC/MS Pos |  | [HMDB11170](http://www.hmdb.ca/metabolites/HMDB11170) | 14253342 |  |
|  | gamma-glutamylleucine | LC/MS Pos |  | [HMDB11171](http://www.hmdb.ca/metabolites/HMDB11171) | 151023 |  |
|  | gamma-glutamylphenylalanine | LC/MS Pos |  | [HMDB00594](http://www.hmdb.ca/metabolites/HMDB00594) | 111299 |  |
| Polypeptide | leu-leu-leu | LC/MS Pos |  |  | 259327 |  |
|  | val-val-val | LC/MS Neg |  |  | 235009 |  |
|  | VGAHAGEYGAEALER* | LC/MS Neg |  |  |  |  |
| Benzoate Metabolism | 3,4-dihydroxybenzoate | GC/MS | [C00230](http://www.genome.jp/dbget-bin/www_bget?cpd+C00230) | [HMDB01856](http://www.hmdb.ca/metabolites/HMDB01856) | 72 |  |
|  | 3-hydroxybenzoate | GC/MS | [C00587](http://www.genome.jp/dbget-bin/www_bget?cpd+C00587) | [HMDB02466](http://www.hmdb.ca/metabolites/HMDB02466) | 7420 |  |
|  | benzoate | GC/MS | [C00180](http://www.genome.jp/dbget-bin/www_bget?cpd+C00180) | [HMDB01870](http://www.hmdb.ca/metabolites/HMDB01870) | 243 |  |
|  | p-aminobenzoate (PABA) | LC/MS Pos | [C00568](http://www.genome.jp/dbget-bin/www_bget?cpd+C00568) | [HMDB01392](http://www.hmdb.ca/metabolites/HMDB01392) | 978 |  |
| Chemical | 1,2-propanediol | GC/MS | [C00583](http://www.genome.jp/dbget-bin/www_bget?cpd+C00583) | [HMDB01881](http://www.hmdb.ca/metabolites/HMDB01881) | 1030 |  |
|  | 2-hydroxyisobutyrate | GC/MS |  | [HMDB00729](http://www.hmdb.ca/metabolites/HMDB00729) | 11671 |  |
|  | 3-aminobutyrate | GC/MS |  |  | 10932 |  |
|  | 3-hydroxypyridine | GC/MS |  |  | 7971 |  |
|  | brilliant blue FCF (blue 1) | LC/MS Neg |  |  | 123671;3540259;109671 |  |
|  | glycolate (hydroxyacetate) | GC/MS | [C00160](http://www.genome.jp/dbget-bin/www_bget?cpd+C00160) | [HMDB00115](http://www.hmdb.ca/metabolites/HMDB00115) | 757 |  |
|  | heptaethylene glycol | LC/MS Pos |  |  | 79718 |  |
|  | hexaethylene glycol | LC/MS Pos |  |  | 17472 |  |
|  | N-methylpipecolate | LC/MS Pos |  |  | 11862129;11286529 |  |
|  | N-propionylmethionine | LC/MS Pos |  |  | 103692 |  |
|  | octaethylene glycol | LC/MS Pos |  |  | 78798 |  |
|  | tetraethylene glycol | LC/MS Pos |  |  | 8200 |  |
|  | trizma acetate | GC/MS | [C07182](http://www.genome.jp/dbget-bin/www_bget?cpd+C07182) |  | 6503 |  |
| Drug | 4-acetamidophenol | LC/MS Pos | [C06804](http://www.genome.jp/dbget-bin/www_bget?cpd+C06804) | [HMDB01859](http://www.hmdb.ca/metabolites/HMDB01859) | 1983 |  |
|  | 4-acetaminophen sulfate | LC/MS Neg | [C06804](http://www.genome.jp/dbget-bin/www_bget?cpd+C06804) | [HMDB59911](http://www.hmdb.ca/metabolites/HMDB59911) | 83939 |  |
|  | atorvastatin (lipitor) | LC/MS Neg | [D00887](http://www.genome.jp/dbget-bin/www_bget?cpd+D00887) | [HMDB05006](http://www.hmdb.ca/metabolites/HMDB05006) | 60823 |  |
|  | gabapentin | LC/MS Pos | [D00332](http://www.genome.jp/dbget-bin/www_bget?cpd+D00332) | [HMDB05015](http://www.hmdb.ca/metabolites/HMDB05015) | 3446;6919078 |  |
|  | hydrochlorothiazide | LC/MS Neg | [C07041](http://www.genome.jp/dbget-bin/www_bget?cpd+C07041) | [HMDB01928](http://www.hmdb.ca/metabolites/HMDB01928) | 3639 |  |
|  | metformin | LC/MS Pos | [C07151](http://www.genome.jp/dbget-bin/www_bget?cpd+C07151) | [HMDB01921](http://www.hmdb.ca/metabolites/HMDB01921) | 4091 |  |
|  | metoprolol | LC/MS Pos | [D02358](http://www.genome.jp/dbget-bin/www_bget?cpd+D02358) | [HMDB01932](http://www.hmdb.ca/metabolites/HMDB01932) | 4171 |  |
|  | metoprolol acid metabolite* | LC/MS Pos |  |  | 62936 |  |
|  | ofloxacin | LC/MS Neg | [C07321](http://www.genome.jp/dbget-bin/www_bget?cpd+C07321) | [HMDB01929](http://www.hmdb.ca/metabolites/HMDB01929) | 4583 |  |
|  | probenecid | LC/MS Neg | [D00475](http://www.genome.jp/dbget-bin/www_bget?cpd+D00475) | [HMDB15166](http://www.hmdb.ca/metabolites/HMDB15166) | 4911 |  |
| Food Component/Plant | 1-methyl-beta-carboline-3-carboxylic acid | LC/MS Neg |  |  | 5406157 |  |
|  | 2,3-butanediol | GC/MS | [C03044](http://www.genome.jp/dbget-bin/www_bget?cpd+C03044) | [HMDB03156](http://www.hmdb.ca/metabolites/HMDB03156) | 262 |  |
|  | 2-aminopimelic acid | GC/MS |  | [HMDB34252](http://www.hmdb.ca/metabolites/HMDB34252) | 101122 |  |
|  | 2-oxindole-3-acetate | LC/MS Pos |  |  | 3080590 |  |
|  | 2-piperidinone | GC/MS |  |  | 12665 |  |
|  | 4-hydroxybenzyl alcohol | GC/MS | [C17467](http://www.genome.jp/dbget-bin/www_bget?cpd+C17467) | [HMDB11724](http://www.hmdb.ca/metabolites/HMDB11724) | 125 |  |
|  | 4-hydroxycyclohexylcarboxylic acid | LC/MS Neg | [C04404](http://www.genome.jp/dbget-bin/www_bget?cpd+C04404) | [HMDB01988](http://www.hmdb.ca/metabolites/HMDB01988) | 151138 |  |
|  | 5-ketogluconate | GC/MS | [C01062](http://www.genome.jp/dbget-bin/www_bget?cpd+C01062) | [HMDB11731](http://www.hmdb.ca/metabolites/HMDB11731) | 5460352 |  |
|  | caffeate | GC/MS | [C01197](http://www.genome.jp/dbget-bin/www_bget?cpd+C01197) | [HMDB01964](http://www.hmdb.ca/metabolites/HMDB01964) | 689043 |  |
|  | curcumin | LC/MS Pos | [C10443](http://www.genome.jp/dbget-bin/www_bget?cpd+C10443) | [HMDB02269](http://www.hmdb.ca/metabolites/HMDB02269) | 969516 |  |
|  | diaminopimelate | GC/MS | [C00666](http://www.genome.jp/dbget-bin/www_bget?cpd+C00666) | [HMDB01370](http://www.hmdb.ca/metabolites/HMDB01370) | 439283 |  |
|  | dihydroferulic acid | LC/MS Neg |  |  | 14340 |  |
|  | enterolactone | LC/MS Neg |  |  | 10685477 |  |
|  | ferulate | LC/MS Neg | [C01494](http://www.genome.jp/dbget-bin/www_bget?cpd+C01494) | [HMDB00954](http://www.hmdb.ca/metabolites/HMDB00954) | 445858 |  |
|  | galacturonate | GC/MS | [C08348](http://www.genome.jp/dbget-bin/www_bget?cpd+C08348) | [HMDB02545](http://www.hmdb.ca/metabolites/HMDB02545) | 84740 |  |
|  | gallate | GC/MS | [C01424](http://www.genome.jp/dbget-bin/www_bget?cpd+C01424) | [HMDB05807](http://www.hmdb.ca/metabolites/HMDB05807) | 370 |  |
|  | genistein | LC/MS Neg | [C06563](http://www.genome.jp/dbget-bin/www_bget?cpd+C06563) | [HMDB03217](http://www.hmdb.ca/metabolites/HMDB03217) | 5280961 |  |
|  | gluconate | GC/MS | [C00257](http://www.genome.jp/dbget-bin/www_bget?cpd+C00257) | [HMDB00625](http://www.hmdb.ca/metabolites/HMDB00625) | 10690 |  |
|  | homostachydrine* | LC/MS Pos | [C08283](http://www.genome.jp/dbget-bin/www_bget?cpd+C08283) | [HMDB33433](http://www.hmdb.ca/metabolites/HMDB33433) | 441447 |  |
|  | indolin-2-one | LC/MS Pos | [C12312](http://www.genome.jp/dbget-bin/www_bget?cpd+C12312) |  | 321710 |  |
|  | Isobar: malitol, cellobiotol, D-lactitol | LC/MS Neg |  |  |  |  |
|  | naringenin | LC/MS Neg | [C00509](http://www.genome.jp/dbget-bin/www_bget?cpd+C00509) | [HMDB02670](http://www.hmdb.ca/metabolites/HMDB02670) | 932 |  |
|  | oleanolate | GC/MS | [C17148](http://www.genome.jp/dbget-bin/www_bget?cpd+C17148) | [HMDB02364](http://www.hmdb.ca/metabolites/HMDB02364) | 10494 |  |
|  | phytanate | GC/MS | [C01607](http://www.genome.jp/dbget-bin/www_bget?cpd+C01607) | [HMDB00417,HMDB00801,HMDB00553](http://www.hmdb.ca/metabolites/HMDB00417,HMDB00801,HMDB00553) | 26840 |  |
|  | piperidine | LC/MS Pos | [C01746](http://www.genome.jp/dbget-bin/www_bget?cpd+C01746) | [HMDB34301](http://www.hmdb.ca/metabolites/HMDB34301) | 8082 |  |
|  | piperine | LC/MS Pos | [C03882](http://www.genome.jp/dbget-bin/www_bget?cpd+C03882) | [HMDB29377](http://www.hmdb.ca/metabolites/HMDB29377) | 638024 |  |
|  | quinate | GC/MS | [C00296](http://www.genome.jp/dbget-bin/www_bget?cpd+C00296) | [HMDB03072](http://www.hmdb.ca/metabolites/HMDB03072) | 6508 |  |
|  | saccharin | LC/MS Neg | [D01085](http://www.genome.jp/dbget-bin/www_bget?cpd+D01085) | [HMDB29723](http://www.hmdb.ca/metabolites/HMDB29723) | 5143 |  |
|  | secoisolariciresinol diglucoside | LC/MS Neg |  |  | 9917980 |  |
|  | sitostanol | GC/MS | [C19644](http://www.genome.jp/dbget-bin/www_bget?cpd+C19644) | [HMDB00494](http://www.hmdb.ca/metabolites/HMDB00494) | 15559396 |  |
|  | solanidine | LC/MS Pos | [C06543](http://www.genome.jp/dbget-bin/www_bget?cpd+C06543) | [HMDB03236](http://www.hmdb.ca/metabolites/HMDB03236) | 65727 |  |
|  | stachydrine | LC/MS Pos | [C10172](http://www.genome.jp/dbget-bin/www_bget?cpd+C10172) | [HMDB04827](http://www.hmdb.ca/metabolites/HMDB04827) | 115244 |  |
|  | sucralose | LC/MS Pos | [C12285](http://www.genome.jp/dbget-bin/www_bget?cpd+C12285) | [HMDB31554](http://www.hmdb.ca/metabolites/HMDB31554) | 71485 |  |
| Xanthine Metabolism | 1,3,7-trimethylurate | LC/MS Neg | [C16361](http://www.genome.jp/dbget-bin/www_bget?cpd+C16361) | [HMDB02123](http://www.hmdb.ca/metabolites/HMDB02123) | 79437 |  |
|  | 1,3-dimethylurate | LC/MS Neg |  | [HMDB01857](http://www.hmdb.ca/metabolites/HMDB01857) | 70346 |  |
|  | 1,7-dimethylurate | LC/MS Neg | [C16356](http://www.genome.jp/dbget-bin/www_bget?cpd+C16356) | [HMDB11103](http://www.hmdb.ca/metabolites/HMDB11103) | 91611 |  |
|  | 1-methylurate | LC/MS Pos | [C16359](http://www.genome.jp/dbget-bin/www_bget?cpd+C16359) | [HMDB03099](http://www.hmdb.ca/metabolites/HMDB03099) | 69726 |  |
|  | 1-methylxanthine | LC/MS Pos | [C16358](http://www.genome.jp/dbget-bin/www_bget?cpd+C16358) | [HMDB10738](http://www.hmdb.ca/metabolites/HMDB10738) | 80220 |  |
|  | 3,7-dimethylurate | LC/MS Neg | [C16360](http://www.genome.jp/dbget-bin/www_bget?cpd+C16360) | [HMDB01982](http://www.hmdb.ca/metabolites/HMDB01982) | 83126 |  |
|  | 5-acetylamino-6-amino-3-methyluracil | LC/MS Neg | [C16366](http://www.genome.jp/dbget-bin/www_bget?cpd+C16366) | [HMDB04400](http://www.hmdb.ca/metabolites/HMDB04400) | 88299 |  |
|  | 7-methylxanthine | LC/MS Pos | [C16353](http://www.genome.jp/dbget-bin/www_bget?cpd+C16353) | [HMDB01991](http://www.hmdb.ca/metabolites/HMDB01991) | 68374 |  |
|  | caffeine | LC/MS Pos | [C07481](http://www.genome.jp/dbget-bin/www_bget?cpd+C07481) | [HMDB01847](http://www.hmdb.ca/metabolites/HMDB01847) | 2519 |  |
|  | paraxanthine | LC/MS Pos | [C13747](http://www.genome.jp/dbget-bin/www_bget?cpd+C13747) | [HMDB01860](http://www.hmdb.ca/metabolites/HMDB01860) | 4687 |  |
|  | theobromine | LC/MS Pos | [C07480](http://www.genome.jp/dbget-bin/www_bget?cpd+C07480) | [HMDB02825](http://www.hmdb.ca/metabolites/HMDB02825) | 5429 |  |
| *indicates compounds that have not been officially confirmed based on a standard, but we are confident in its identity | | | | | |  |

**Supplemental Figure Legends**

Supplemental Figure S1.

**Diagram of tissue and stool metabolites distinguished by microbial or mammalian production**. Metabolites shown in red are produced exclusively or mainly through microbial metabolism. Metabolites shown in blue are produced exclusively or mainly through mammalian metabolism. Note: 1 = Tissue detection only and 2 = stool detection only. This conceptual framework is adapted from [[1](#_ENREF_1)] which explores the metabolites modulated by gut bacteria activities.

**Supplemental Figure S1.** **
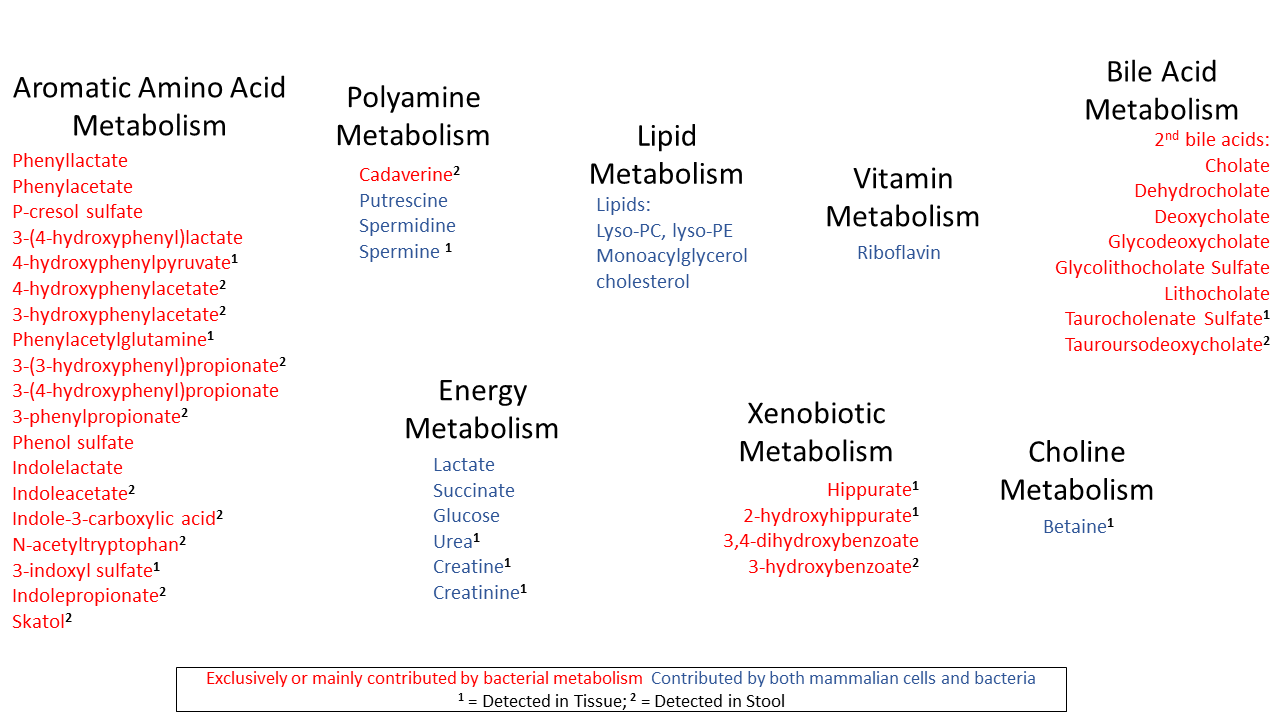
**

[1] Guo, L., Milburn, M. V., Ryals, J. A., Lonergan, S. C.*, et al.*, Plasma metabolomic profiles enhance precision medicine for volunteers of normal health. *Proceedings of the National Academy of Sciences of the United States of America* 2015, *112*, E4901-4910.
